# Quantification of transplant-derived circulating cell-free DNA in absence of a donor genotype

**DOI:** 10.1371/journal.pcbi.1005629

**Published:** 2017-08-03

**Authors:** Eilon Sharon, Hao Shi, Sandhya Kharbanda, Winston Koh, Lance R. Martin, Kiran K. Khush, Hannah Valantine, Jonathan K. Pritchard, Iwijn De Vlaminck

**Affiliations:** 1 Department of Genetics, Stanford University, Stanford, California, United States of America; 2 Department of Biology, Stanford University, Stanford, California, United States of America; 3 Meinig School of Biomedical Engineering, Cornell University, Ithaca, New York, United States of America; 4 Pediatric Stem Cell Transplantation, Lucille Packard Children's Hospital, Stanford, California, United States of America; 5 Howard Hughes Medical Institute, Stanford University, Stanford, California, United States of America; 6 Departments of Bioengineering and Applied Physics, Stanford University, Stanford, California, United States of America; 7 Division of Cardiovascular Medicine, Stanford University School of Medicine, Stanford, California, United States of America; 8 National Institutes of Health, Bethesda, Maryland, United States of America; Princeton University, UNITED STATES

## Abstract

Quantification of cell-free DNA (cfDNA) in circulating blood derived from a transplanted organ is a powerful approach to monitoring post-transplant injury. Genome transplant dynamics (GTD) quantifies donor-derived cfDNA (dd-cfDNA) by taking advantage of single-nucleotide polymorphisms (SNPs) distributed across the genome to discriminate donor and recipient DNA molecules. In its current implementation, GTD requires genotyping of both the transplant recipient and donor. However, in practice, donor genotype information is often unavailable. Here, we address this issue by developing an algorithm that estimates dd-cfDNA levels in the absence of a donor genotype. Our algorithm predicts heart and lung allograft rejection with an accuracy that is similar to conventional GTD. We furthermore refined the algorithm to handle closely related recipients and donors, a scenario that is common in bone marrow and kidney transplantation. We show that it is possible to estimate dd-cfDNA in bone marrow transplant patients that are unrelated or that are siblings of the donors, using a hidden Markov model (HMM) of identity-by-descent (IBD) states along the genome. Last, we demonstrate that comparing dd-cfDNA to the proportion of donor DNA in white blood cells can differentiate between relapse and the onset of graft-versus-host disease (GVHD). These methods alleviate some of the barriers to the implementation of GTD, which will further widen its clinical application.

## Author summary

More than 180,000 people live with organ transplants in the US. Monitoring the health of the allograft is a critically important component of post-transplant therapy. Recently, we have demonstrated that cell-free DNA (cfDNA) enables diagnosis of post-transplant rejection. In this approach, genotyping of the recipient and the donor together with shotgun sequencing of cfDNA are used to quantify the proportion of donor-derived cfDNA (dd-cfDNA). This approach offers key advantages over the current practice of organ biopsy, which is invasive, costly, and risky. However, the difficulty and cost of establishing a pure reference donor genotype is a major barrier to its widespread clinical implementation. Here, we developed a computational approach that enables an accurate estimation of dd-cfDNA levels without the need for a donor genotype. In addition, we provide a solution of closely related recipients and donors—a scenario which is common in bone marrow and kidney transplants—by accounting for the identity-by-descent between the recipient and the donor. Last, we demonstrate a new application of cfDNA in the noninvasive diagnosis of Graft Versus Host Disease, a serious and difficult-to-diagnose complication of allogeneic bone marrow and stem cell transplantation.

## Introduction

Solid-organ transplantation is now a common practice [1]. However, the clinical outcomes remain poor with median survival rate of 5.3 years for lung and 11 years for heart [2–4]. Accurate monitoring of allograft health is essential for long-term survival of the transplant recipient. The current gold standard method of allograft rejection surveillance is the biopsy (transbronchial biopsy for lung transplant and endomyocardial biopsy for heart transplant), but this invasive technique suffers from high cost and myriad complications [5,6]. Recently, enumeration of cell-free, donor-derived DNA (dd-cfDNA) in the transplant recipient plasma using shotgun sequencing was suggested as a tool to monitor organ health [7–9]. For female recipients of a male graft, it is relatively straightforward to identify and quantify donor specific cfDNA through molecular assays targeting Y chromosome DNA [7]. Genome Transplant Dynamics (GTD) quantifies donor-derived DNA regardless of the sex of the transplant donor or recipient. GTD takes advantage of single-nucleotide polymorphisms (SNPs) distributed across the genome to discriminate donor and recipient DNA molecules. This concept was first demonstrated in a retrospective study in heart transplantation [10], where increased levels of donor-derived DNA were shown to correlate with acute cellular rejection (ACR) events as determined by endomyocardial biopsy.

However, the current implementation of shotgun based GTD, which we will refer to as the “two-genomes” method, requires genotyping of both the donor and recipient. In contrast to the recipient genotype that is easy to obtain, the donor genotype is often unavailable. We therefore set out to develop a method that enables dd-cfDNA monitoring using shotgun sequencing without donor genotype information—a “one-genome” method. We apply the method to lung and heart transplant recipient cohort data and demonstrate that the performance of a one-genome method approaches the performance of the two-genomes method.

As in solid organ, cfDNA examination may inform about the status of a bone marrow transplant. More than 10,000 patients receive life-saving allogeneic bone or stem cell transplants in the United States each year, yet complications due to acute Graft-Versus Host Disease (GVHD) occur frequently (in up to 50% of patients), cause morbidity and mortality, and limit the therapeutic value of allogeneic bone or stem cell transplants [11]. Diagnosis of GVHD currently relies on invasive biopsy procedures, such as skin biopsy, colonoscopy, upper endoscopy or even liver biopsy. These are painful, burdensome, expensive and potentially dangerous procedures in these profoundly immunocompromised patients. However, few studies have examined the utility of cfDNA in the context of bone marrow or stem cell transplantation and cfDNA was not to monitor GTD of bone marrow transplant [12].

As opposed to heart and lung transplants, in which the donor and recipient are not related, in bone marrow transplantation (and other transplants, such as kidney) close relations are common. Therefore, we refined the “one-genome” approach to robustly handle the scenario where the donor and recipient share a recent common ancestor. Chromosomes of such donor-recipient share long stretches of DNA that are Identical By Descent (IBD) [13], which may lead to underestimation of dd-cfDNA. To solve this, we use a hidden Markov model (HMM) of local IBD states along the genome. We examined whether our “one-genome” GTD approach can be applied to analyze complications in post bone marrow transplant patients (8 patients, 76 samples). We show that our “one-genome” approach, which integrate over IBD states, and the previous “two-genomes” approach give similar estimations of cfDNA in bone marrow transplant patients. In addition, we suggest that a comparison of the fraction of dd-cfDNA in plasma and in the cellular fraction can be used to discriminate graft loss or relapse, which are accompanied by an increase in recipient cfDNA from blood cells, from GVHD, which is accompanied by an increase in recipient cfDNA from other recipient tissues.

## Results

### Quantifying dd-cfDNA in lung and heart transplant recipients

**Fig 1:**
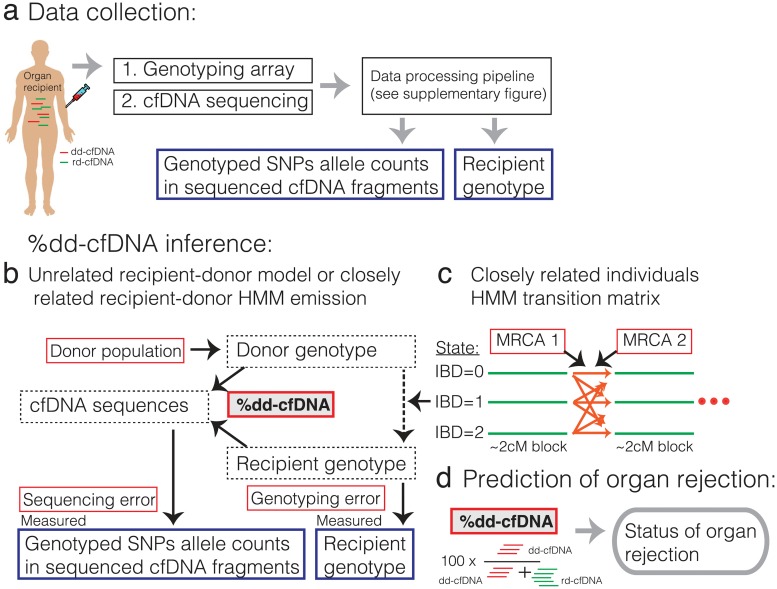
Illustration of our approach. (a) A blood sample is used to genotype the recipient (cellular fraction, done once) and to sequence the cfDNA (see S1 Fig for details). (b-c) Illustration of the “one-genome” statistical model for inferring the percent of dd-cfDNA (red box with gray background). Black arrows show statistical dependency and text boxes show nuisance parameters (red box with white background), hidden variables (dotted line box) and measured data (blue box). (b) Shows the model which assumes that the donor and the recipient are unrelated. (c) When donor and recipient may be closely related (in this work, in case of a bone marrow transplant) the donor genotype depends on the recipient genotype and the local identity by descent (IBD) state between the recipient and donor genotypes. IBD states are modeled for each block of ~2cM along the genome. Transitions between IBD states depend on the number of meioses that separate each pair of recipient-donor chromosomes given their most recent diploid common ancestor (MRCA 1 and MRCA 2). (d) the inferred percent of dd-cfDNA is used to predict organ rejection.

We developed a statistical model that quantifies donor- and recipient-derived cfDNA fragments in the absence of donor genotype information (Fig 1, see Methods for a formal description of the model). To quantify the observed abundance of alleles of each genotyped SNP in cfDNA sequences (Fig 1a, S1 Fig) [8], we first filter low quality reads, reads that are not mapped uniquely to the genome, and reads with potential for mapping biased by genetic variability [14]. We then remove duplicated reads and count allele appearances of each genotyped SNP (SAMtools mpileup function [15]). We use all genotyped SNPs, as opposed to the “two genomes” method that uses only SNPs that are homozygous but differ between recipient and donor. The observed allele appearances in cfDNA and the recipient genotype are the inputs for our “one-genome” model.

To calculate the probability of the observed cfDNA, we first calculate the probability of each possible donor and recipient genotype. The likelihood of the recipient genotype is a function of the measured genotype and the genotyping error rate. Vital organ transplants are rarely closely related. Therefore, for heart and lung transplants, our model assumes that the donor genotype is randomly selected from a human population. Given this assumption, the probability of a specific donor allele is its frequency in the population. Our algorithm iterates over 1000 Genomes Project populations and super-populations [16] to detect the most likely ancestral population of the donor. This simplified model achieves satisfying performance in lung and heart transplant, but requires refinement for handling bone marrow transplants in which donor and recipients are often related.

Putting it together, the probability of observing a specific allele in a cfDNA fragment is computed by integrating over all possible recipient and donor genotypes and depends on the sequencing error rate, the fraction of dd-cfDNA in the recipient plasma and the probabilities of observing the allele conditioning on it being donor- or recipient-derived (Fig 1b). Finally, we compute the log-likelihood of the data by summing log-likelihoods over all SNPs, assuming SNPs are independent (this assumption is also made by the two-genomes method). We use an optimization algorithm to find the maximum likelihood parameter values [17].

### Performance of lung and heart rejection predictions

**Fig 2:**
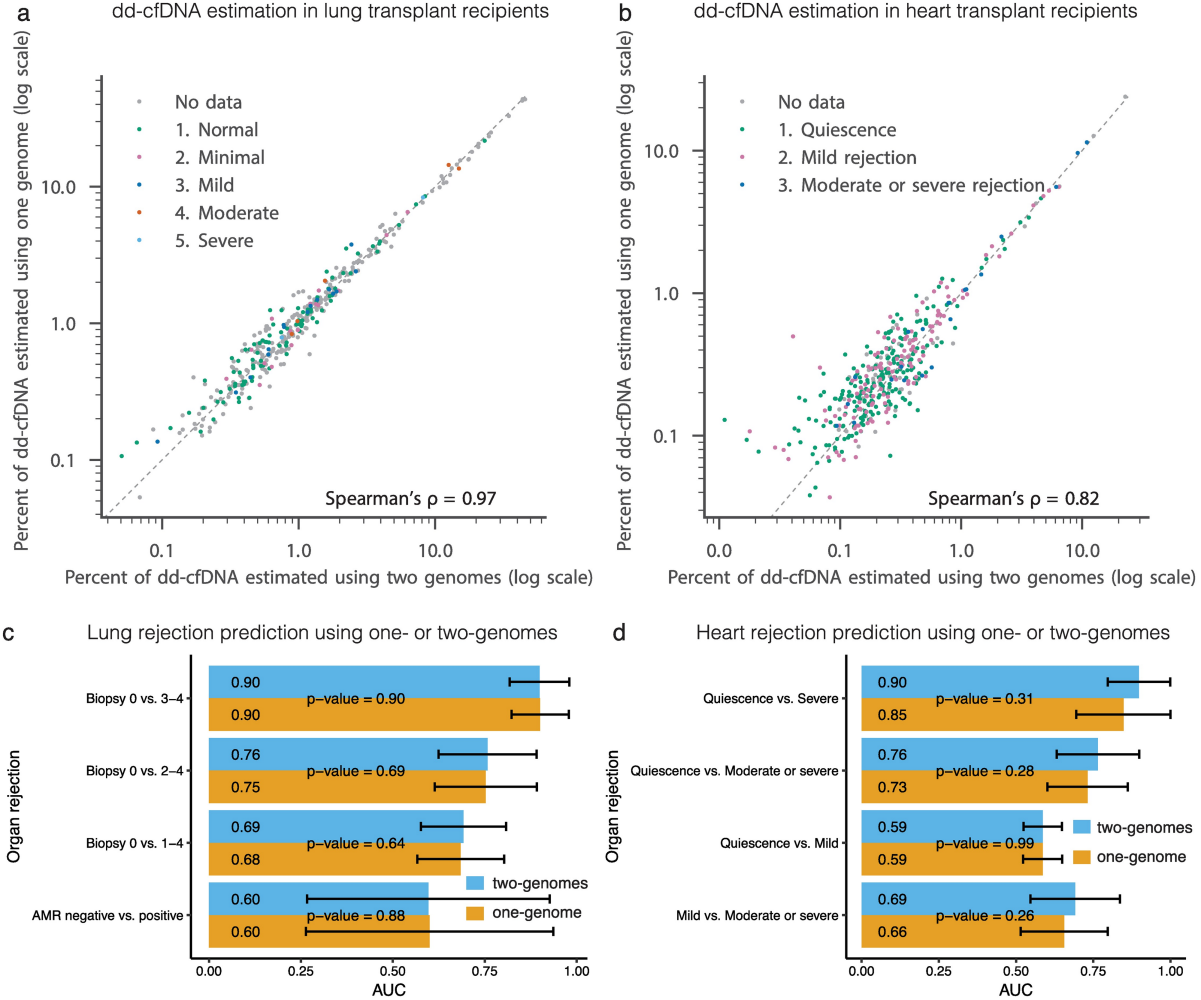
Comparison of predicted levels of dd-cfDNA by one- and two-genomes methods in heart and lung transplant recipients. (a) and (b) Comparison between levels of dd-cfDNA predicted by the two-genomes method (x-axis) and the one-genome method (y-axis). (c) and (d) show a comparison of one- and two-genomes methods predictability of organ rejection. Each bar shows the area under the curve (AUC) of discriminating between two rejection states as measured using biopsies using dd-cfDNA fraction estimates [8,9]. Error bars marks AUC 95% confidence interval. The significance of the difference between corresponding receiver operating characteristic (ROC) of the one-genome and two-genomes was evaluated using the DeLong two-sided test [31,32].

To assess the performance of the one-genome model, we directly compared estimates of dd-cfDNA for the one and two-genome methods. We find that the dd-cfDNA predictions based on the two models are highly correlated for lung transplants (51 patients, 382 samples, Pearson’s R^2^ = 0.996, Spearman’s ρ = 0.97, mean absolute error = 0.002; Fig 2a, S2 Fig; S1 Table). For heart transplant recipients (59 patients, 435 samples), dd-cfDNA level estimates resulting from both methods were also highly correlated, but not as strongly as in the lung cohort (Pearson’s R^2^ = 0.990, Spearman’s ρ = 0.82, mean absolute error = 0.001; Fig 2b; S2 Table). This is due to the lower levels of dd-cfDNA in heart cohort make the inference harder; as reflected by the increase in the difference between the predictions of the two methods relative to the predicted value as dd-cfDNA levels decrease below 0.5% (S2 Fig).

We next compared the performance in the diagnosis of organ rejection based on the one- and two-genomes estimated levels of dd-cfDNA (Fig 1d). We found that the two methods perform similarly in differentiating between different levels of organ rejection as determined by biopsy, for both heart and lung transplants (Fig 2c and 2d, differences in detection quality are not significant, DeLong two-sided test). We note that for the two-genome method, it is possible to measure the assay error rate independently, by analyzing SNPs for which the donor and recipient genotypes are matched and homozygous. This measure can then be used to perform a background error-correction as described previously [8]. We found this error-correction slightly improves the detection quality for the two-genomes method in the heart cohort (S3 Fig). Error correction may improve the performance of the one-genome method to similar extent. In principle, this can be done using pre transplant cfDNA samples by comparing homozygote sites to the sequencing reads (pre transplant samples from heart and lung transplants were not available to this study). We conclude that donor genotyping is not required for lung transplant recipients. Donor genotyping can also be avoided in heart transplant recipients, but the accuracy of the test may be reduced slightly, in particular in detecting moderate rejection.

### Quantifying donor-derived cfDNA in bone marrow transplant recipients

Because bone marrow donors are often close relatives of the recipients, the assumption that the donor is randomly selected from the population no longer holds. Chromosomes of closely-related individuals contain long segments of identical genotype. These segments are said to be identical by descent (IBD). The abundance and length of the IBD segments depend on the number of meioses separating the two chromosomes and the recombination rate [13,18–21]. Ignoring IBD may lead to under-estimation of dd-cfDNA level. We therefore extended our model to account for possible IBD by learning recipient-donor relatedness. We implemented a Hidden Markov Model (HMM) with three states (Fig 1b and 1c; see Methods for details): when there is no IBD (IBD = 0), the model emission probabilities are similar to the above unrelated donor-recipient model; when one pair of chromosomes is IBD (IBD = 1), the genotype of one donor allele will be similar to one of the recipient alleles and the other donor allele likelihood depends on its abundance in the population (independently of the recipient genotype); lastly, when both chromosome pairs are in IBD (IBD = 2) the recipient and donor genotypes are identical. In our model, transitions between IBD states can occur only between pre-calculated 2centimorgan blocks [22]. Transition probabilities depend on the recipient-donor relatedness, which is represented by the number of meioses separating each pair of donor-recipient chromosomes (Fig 1b). In other words, in our refined model, the donor genotype depends on the population allele frequency and the recipient genotype according to the local IBD state.

### Accuracy of dd-cfDNA level estimations in bone marrow transplant recipients

**Fig 3:**
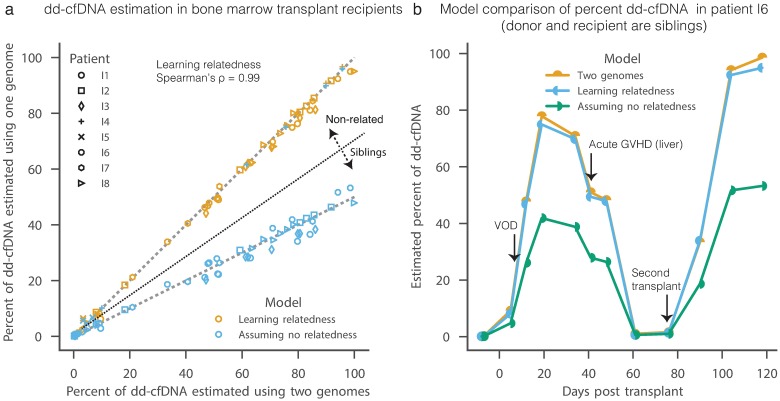
Comparison of predicted levels of dd-cfDNA by one- and two-genomes methods in bone marrow transplant recipients. (a) Comparison between levels of dd-cfDNA predicted by the two-genomes method (x-axis) and the one-genome method (y-axis) when learning donor and recipient relatedness (orange) or naively assuming that they are unrelated (blue). The later under-estimates dd-cfDNA levels when the recipient and donor are siblings. Dashed lines show 1:1 and 2:1 ratios. (b) An example of cfDNA level estimates in a single bone marrow transplant recipient that is a sibling of the donor (I6).

To evaluate the performance of the refined one-genome method, we applied it to 76 samples from 8 bone marrow transplant recipient patients (Fig 3, S2 Fig; S3 Table). Two of the donors (for patients I4 and I5) were unrelated to the recipients and six were siblings of the recipients (S4 Table). As expected, the naïve implementation of the one-genome method underestimates dd-cfDNA in sibling donors, that share about 50% of their genotype due to IBD, but not in unrelated donors (Fig 3a). When our model is set to learn the relationship between the donor and the recipient, its dd-cfDNA level estimates match the two-genomes method (Pearson’s R^2^ = 0.998, Spearman’s ρ = 0.99, mean absolute error = 0.004; Fig 3a). Reassuringly, these predictions strongly correlate with the fraction of reads originating from the X chromosomes when the donor and recipient sex is different (S4 Fig). We conclude that accurate estimation of dd-cfDNA in bone marrow recipients does not require donor genotyping. These results may also apply to other settings, such as kidney transplants.

### Differentiating between relapse and graft versus host disease in bone marrow transplant recipients

The success of bone marrow transplants is often impaired by cancer relapses and graft versus host disease (GVHD) [11]. Diagnosing and differentiating between the two remains a major challenge in the field. The current gold standard for a successful engraftment is absolute neutrophil count greater than 500 for three consecutive days. This corresponds to 47–82% dd-cfDNA in our patients (S5 Fig; S4 Table). We notice that in patients who relapse (patients I3) or have acute GVHD (patients I1 and I8) or chronic GVHD (patients I2), the level of cfDNA drops after reaching its peak (24%, 33%, 11% and 24%, respectively). Although our cohort is too small to assess significance, this observation suggests that GTD can be used to monitor bone marrow transplant health.

**Fig 4:**
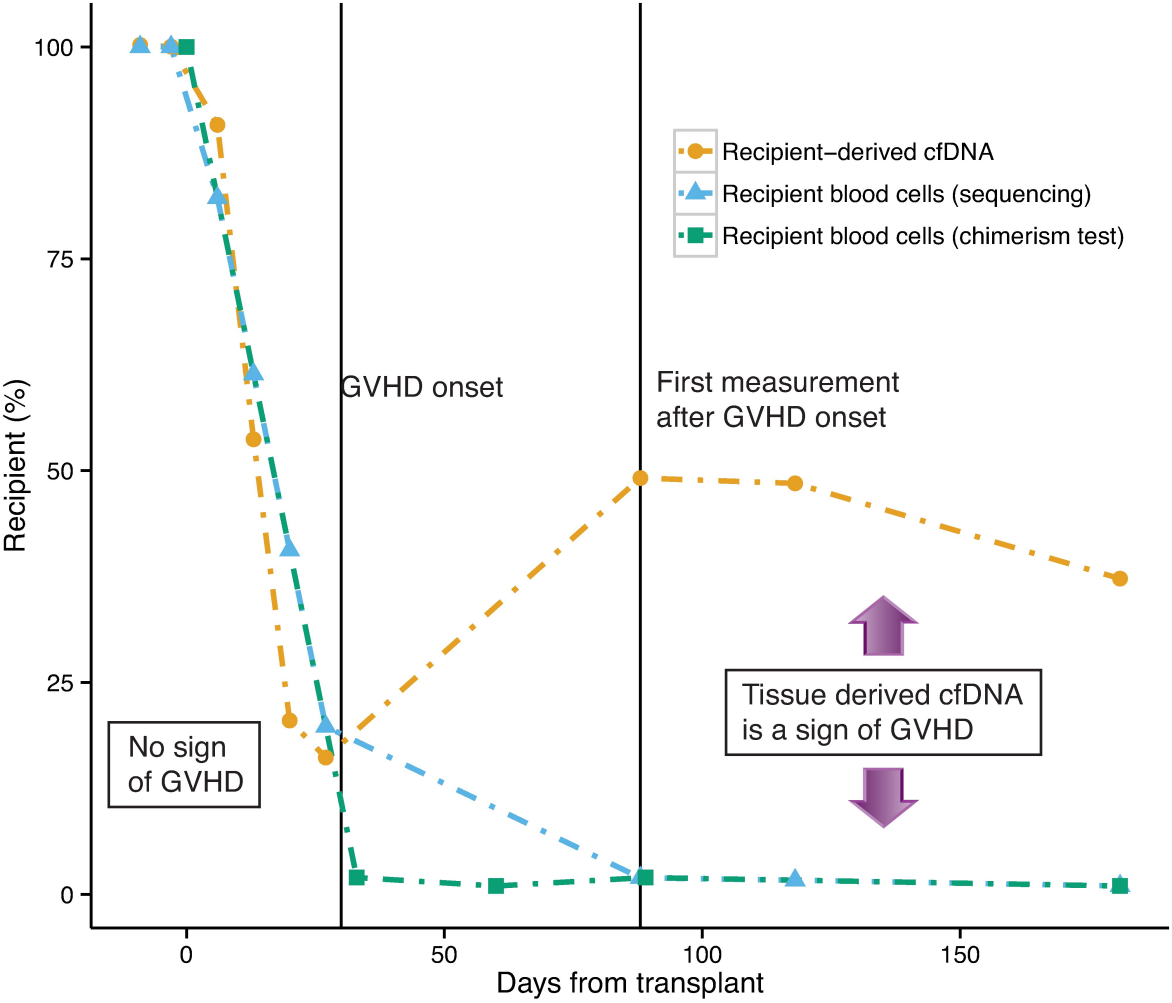
Comparing the fraction of cfDNA that is recipient-derived to the fraction of recipient-derived blood cells may detect GVHD. A proof of principle in a single bone marrow transplant recipient (patient I8), that differences in the recipient-derived cfDNA and recipient-derived blood cells levels may indicate onset of GVHD. The difference between the two measurements is due to injured tissue-derived cfDNA. In contrast, when relapse occurs both measurements should show an increase in recipient-derived fraction (not shown in this figure). This may help to distinguish between GVHD and relapse in bone-marrow transplanted patients.

What are potential explanations for an increase in the level of cfDNA from recipient origin? In the case of a cancer relapse, the fraction of lymphocytes of recipient origin increases. The cfDNA will therefore reflect increasing levels of recipient-origin lymphocytes. On the other hand, in the case of GVHD, the fraction of lymphocytes from recipient origin does not increase. In this case, the increase in dd-cfDNA is caused by injury to recipient tissues. We therefore hypothesized that differences in the recipient-origin DNA in the cellular and plasma (cell-free) fractions can distinguish between relapse and GVHD. As a proof of principle, we sequenced both the cfDNA and the cellular fraction in patient I8. In agreement with our hypothesis, the two values match until the onset of the acute GVHD (since most cfDNA originates from lymphocytes) and then diverge—after the onset of GVHD, the cellular fraction remains low and cfDNA level increases (Fig 4). This “N of one” experiment demonstrates the great potential of GTD to distinguish between relapses and GVHD—an urgent unmet need in the field.

### Method performances in various simulated levels of dd-cfDNA, genotyped SNPs and cfDNA sequencing depth

To further assess the robustness and accuracy of the one-genome method described here, we performed extensive simulations (S6–S11 Figs; S5 Table). To this end, we created synthetic mixtures of two individuals using data of pre-transplant cfDNA available from the bone-marrow transplant cohort (patients I8, I2, I3). We simulated degrees of donor-recipient relatedness using the same approach as was used for IBD inference and sub-sampled both the SNPs and the cfDNA sequencing reads (see Methods for details). We compared the simulated level of dd-cfDNA to the level that was estimated by a one-genome model that learns the relatedness between the donor and the recipient.

In general, we found an excellent agreement between the simulated and predicted dd-cfDNA across relatedness (Spearman’s ρ >0.997 and median absolute error 0.0006–0.0012 using 600-700K SNPs, and 1.75x cfDNA sequencing; S6 Fig). In the range of 0.2%-10% that is clinically important for heart and lung transplant rejection prediction, the error is usually below 1–5% of the predicted value. The performances reduced only slightly when 1x cfDNA sequencing and 600-700K or 157K SNPs are used as input (S7–S9 Figs). We further evaluated the robustness of the one-genome method against the depth of cfDNA sequencing (S10 Fig) and the number of genotyped SNPs (S11 Fig). We find even when only 150K SNPs are used or a 0.33x cfDNA sequencing (~40 USD in our academic sequencing center) the relative absolute error is within 10% of the simulated value (for example absolute error of 0.1% for 1% simulated dd-cfDNA). For bone marrow transplants, even the lowest cfDNA sequence coverage that we simulated (0.1x for unrelated and 1x for related) gave very good results (Spearman’s ρ > 0.993). The relative error increases below 0.1% dd-cfDNA but the absolute error is very low in that range (with 600K SNPs and 1.75x it is most often below 0.01% and at most 0.1%). In summary, our simulations show that inference of the percent of dd-cfDNA using the one-genome method has low error across the clinically relevant range.

## Discussion

In conclusion, we have introduced several technical improvements to shotgun sequencing-based GTD that alleviate barriers to its widespread clinical implementation, including the difficulty and cost of establishing a pure reference donor genotype. We provide a method to quantify dd-cfDNA in plasma in the absence of donor genotype information and demonstrate that its diagnostic performance approaches the diagnostic performance of conventional GTD. We extend the method with integration over hidden local IBD states to be able to handle the challenging scenario in which the transplant donor and transplant recipient are close relatives—a scenario that frequently arises in kidney and bone marrow transplantation. We note that the problem of estimating dd-cfDNA levels is related to identifying contamination in a sequenced sample for which the genotype is known [23], this application may also benefit from our solution for close relatives.

Very recently, targeted-sequencing was suggested as an alternative method to assess dd-cfDNA levels [24]. However, this method suffers from a relatively high lower limit of detection (0.2% dd-cfDNA). The method furthermore has a relatively low upper limit of detection (25% dd-cfDNA) which excludes application in liver, lung and bone marrow transplantation [9,24,25]. Shotgun sequencing GTD has a larger dynamic range and is furthermore simultaneously informative of infection [9,26] and tissues-of-origin of cfDNA [27]. Detailed simulations indicate that donor DNA levels can be robustly captured using shotgun sequencing, in absence of a donor genotype, even with low coverage sequencing and genotyping. An analysis of the accuracy of donor DNA predictions as function of depth of sequencing indicated that 0.33–0.5x cfDNA sequence coverage of the human genome may be sufficient to robustly quantify donor DNA. Such coverage can be achieved at a cost of 40–60 USD, at our academic genomics facility. We note that, at such low coverage, the cost of sequencing is a minor component of the overall assay cost.

Last, we introduce a new application of cell-free DNA in the monitoring of the health and success of bone marrow transplants. We show that the proportion of donor DNA in plasma can be used to monitor the success of engraftment in allogeneic bone marrow transplantation, and we provide evidence demonstrating that combined measurement of chimerism in the cell-free and cellular compartments can distinguish changes in dd-cfDNA due to graft loss and GVHD. Collectively, these studies greatly expand the utility of GTD in transplantation.

## Methods

### cfDNA sequencing and genotype data collection

The cfDNA sequencing and genotyping data for heart and lung transplant recipients was available from our previous studies [8,9]. Additional dd-cfDNA measurements were performed for bone marrow transplant patients (8 patients, 76 samples), using methods previously described [8,9]. In short, recipient plasma was collected at several time points before the transplant procedure (two time points) and at several time points after transplantation sequenced. cfDNA was purified from plasma and sequenced (Illumina HiSeq 200 or HiSeq 2500 1 × 50bp or 2 × 100bp). Donor and recipient genotyping was performed using Illumina whole-genome arrays HumanOmni2.5–8 or HumanOmni1 prior to the transplant.

### Estimating allele representation in cfDNA fragments

Several steps were applied to the cfDNA sequencing reads to achieve counts of allele representation for each genotyped SNP (S1 Fig). First, low quality reads were filtered out (reads in which more than 50% of the base qualities are below 20). Second, reads were mapped to the human genome (UCSC version hg19) using bowtie2 [28] (with the following parameters: -D 20 -R 3 -N 0 -L 20 -i S,1,0.50 -I 20 -X 500—no-mixed—no-discordant—no-unal–t) and SAMtools [29] was used to filter paired ends reads where one of the reads was unmapped (flags -f 3 -F 3852 for pair ends reads and -F 3844 for single end reads) or reads with P>0.05 to be mapped non-uniquely. Third, WASP [14] was applied to remove reads in which the mapping may be biased by the genotype. Fourth, duplicated reads (reads that map to the same exact location) were removed by scripts that selects randomly which of the duplicated reads to keep and are therefore not biased towards a specific genotype[14]. Fifth, chromosomal coverage was computed using HTSeq [30]. Sixth, the number of cfDNA reads that contain each SNP allele was computed using SAMtools mpileup function. These counts were used as input for the model.

### Estimating cfDNA donor-derived in recipient that is unrelated to the donor

As vital organs such as heart and lungs are donated post-mortem, donors are usually unrelated to recipients. Therefore, our model assumes that the donor was randomly selected from some ancestral population. This is clearly a simplifying assumption—donors may have a mixed ancestry and their MHC is often matched to the recipient MHC—nonetheless we find that we can achieve good performance by making this assumption (we note that modeling of mixed ancestry did not improved the predictions). Given the population from which the donor was drawn, the prior probability of observing each allele in the donor is exactly the allele frequency in the population (assuming Hardy-Weinberg equilibrium). Since the donor population is unknown, the optimization function iterates over 1000 Genomes project populations and super-population [16] and selects the population that maximize the likelihood. The goal of the model is to estimate the fraction of cfDNA that is donor-derived (dd-cfDNA) given the recipient measured genotype and the cfDNA reads (see model illustration in Fig 1b).

Formally, let N be the number of bi-allelic SNP that were genotyped in the recipient; A and B denote the two possible alleles for SNP_i_ where *i* ∈ {1,2,…,*N*}; $\left(R_{i_{1}},R_{i_{2}}\right)$ be the recipient true genotype in SNP_i_; $\left(R_{i_{1}}^{*},R_{i_{2}}^{*}\right)$ be the recipient observed (measured) genotype in SNP_i_; $\left(D_{i_{1}},D_{i_{2}}\right)$ be the donor true genotype in SNP_i_; $f_{i_{A}}^{pop_{m}}$ be the frequency of allele A of SNP_i_ in population m; $C_{i_{j\in \left\{1,2,\ldots ,K_{i}\right\}}}$ be the true SNP_i_ allele in a cfDNA fragment that contains it; and $C_{i_{j\in \left\{1,2,\ldots ,K_{i}\right\}}}^{*}$ be the observed allele of SNP_i_ from a sequencing read of this fragment. The observed data (R*, C*) is therefore the recipient measured genotype at N SNPs and the observed allele of these SNPs in cfDNA sequencing reads.

Lets also define the following model parameters (θ): *d* ∈ [0,1] is the fraction cfDNA fragments that are donor-derived (dd-cfDNA); *e*_*s*_ ∈ [10^−9^, 10^−2^] is the sequencing error rate; *e*_*g*_ ∈ [10^−9^, 10^−3^] is the genotyping error rate; and *Pop*_*m*_ ∈ {1,…,*M*} is one of M ancestral population and super populations of 1000 genomes project from which the donor is randomly drawn. The model sequencing and genotyping error rates were bound to technically realistic range. The goal of our model is to estimate *d*–the fraction of dd-cfDNA.

In our model, of the dependency of the observed recipient genotype of SNP_i_ on the true genotype involves the genotyping error rate. So, for example:

$$\begin{array}{ll} P\left(\left(R_{i_{1}}^{*},R_{i_{2}}^{*}\right)=AA\text{|}(R_{i_{1}},R_{i_{2}}\text{)};e_{g}\right)= & {\left(1-e_{g}\right)}^{2}1\left\{R_{i_{1}}=A,R_{i_{2}}=A\right\}\\ & +2\left(e_{g}\right)\left(1-e_{g}\right)1\left\{R_{i_{1}}=A,R_{i_{2}}=B\right\}\\ & +{\left(e_{g}\right)}^{2}1\left\{R_{i_{1}}=B,R_{i_{2}}=B\right\} \end{array}$$

Similarly, the dependency of the observed allele in a sequencing read that map to SNP_i_ on the true allele of SNP_i_ in the cfDNA fragment that was sequenced involves the sequencing error rate (this also capture PCR amplifications errors):

$$P\left(C_{i_{j}}^{*}=A|C_{i_{j}};e_{s}\right)=\left(1-e_{s}\right)1\left\{C_{i_{j}}=A\right\}+\left(e_{s}\right)1\left\{C_{i_{j}}=B\right\}$$

Following the assumption that the donor was randomly drawn from a population, the genotype of SNP_i_ depends on SNP_i_ alleles frequencies in the population and therefore on which ancestral population is used to achieve the SNP_i_ alleles frequencies estimates:

$$P\left(D_{i_{1}}=d_{1},D_{i_{2}}=d_{2}|pop_{m}\right)=\;f_{i_{d_{1}}}^{pop_{m}}*f_{i_{d_{2}}}^{pop_{m}}\;,\text{ where }d_{1}\in \left\{A,B\right\}\text{ and }d_{2}\in \left\{A,B\right\}$$

Lastly, the probability that a cfDNA sequence that maps to SNP_i_ contains a specific allele of SNP_i_ depends of the true genotypes of the recipient and the donor and the fraction of dd-cfDNA (*d*); for example:

$$\begin{array}{ll} P\left(C_{i_{j}}=A\text{|}(R_{i_{1}},R_{i_{2}}\text{)},(D_{i_{1}},D_{i_{2}});d\right)= & d*\left(\begin{array}{c} 1*1\left\{D_{i_{1}}=A,D_{i_{2}}=A\right\}+\\ 0.5*1\left\{D_{i_{1}}=A,D_{i_{2}}=B\right\}+\\ 0*1\left\{D_{i_{1}}=B,D_{i_{2}}=B\right\} \end{array}0.5*1\left\{D_{i_{1}}=B,D_{i_{2}}=A\right\}+\right)+\\ & \left(1-d\right)*\left(\begin{array}{c} 1*1\left\{R_{i_{1}}=A,R_{i_{2}}=A\right\}+\\ 0.5*1\left\{R_{i_{1}}=A,R_{i_{2}}=B\right\}+\\ 0*1\left\{R_{i_{1}}=B,R_{i_{2}}=B\right\} \end{array}0.5*1\left\{R_{i_{1}}=B,R_{i_{2}}=A\right\}+\right) \end{array}$$

Putting it together the likelihood of observing the recipient genotype and the sequencing reads that map to SNP_i_ is:

$$P_{i}\left(\left(R_{i_{1}}^{*},R_{i_{2}}^{*}\right),\;C_{i_{1}}^{*},\;\ldots \;C_{i_{K_{i}}}^{*}|d,e_{s},e_{g,}pop_{m}\right)\;=\;\prod_{j=1}^{K_{i}}\sum_{c,r_{1},r_{1},d_{1},d_{2,c}\in \left\{A,B\right\}}\left(\begin{array}{c} P\left(C_{i_{j}}^{*}|C_{i_{j}}=c;s_{e}\right)\\ {}*P\left(C_{i_{j}}\text{|}(R_{i_{1}},R_{i_{2}}\text{)},(D_{i_{1}},D_{i_{2}}\right);d)\\ {}*P\left(D_{i_{1}}=d_{1},D_{i_{2}}=d_{2}|pop_{m}\right)\\ {}*P\left(R_{i_{1}}=r_{1},R_{i_{2}}=r_{2}\right)\\ {}*P\left(R_{i_{1}}^{*},R_{i_{2}}^{*}|R_{i_{1}}=r_{1},R_{i_{2}}=r_{2};g_{e}\right) \end{array}\right)$$

Although it is possible to model the probability of the recipient genotype ($P\left(R_{i_{1}},R_{i_{2}}\right)$) using population allele frequency data, we assume here a uniform probability since, in practice the genotyping error is very low and therefore the measured recipient genotype is highly informative on the true recipient genotype.

Finally, assuming that SNPs are independent (this is reasonable assumption because we used only genotyped SNPs), the likelihood function is:

$$L\left(R^{*},C^{*}|\theta \right)=\prod_{i=1}^{N}P\left(\left(R_{i_{1}}^{*},R_{i_{2}}^{*}\right),\;C_{i_{1}}^{*},\;\ldots \;C_{i_{K_{i}}}^{*}|d,s_{e},g_{e,}pop_{m}\right)$$

where R*, C* are genome-wide measured recipient genotype and all mapped sequencing reads correspondingly. We use L-BFGS-B to minimize the negative log likelihood for each possible donor ancestral population and select the population that obtains the minimal negative log likelihood.

### Estimating donor-derived cfDNA in related recipient and donor

In contrast to lung and heart, bone marrow and other organs such as kidney, are often donated by individuals that are closely related to the recipient. Therefore, the assumption that the donor is drawn randomly from the population is no longer valid. Closely-related individuals share stretches of identical haplotypes that were inherited from a recent common ancestor, a phenomenon known as Identity By Descent (IBD). For each pair of chromosomes, IBD segments’ length distribution and total length depend on the number of meioses from their Most Recent Common Ancestor (MRCA). The model accounts for IBD using a non-homogenous Hidden Markov Model (HMM) in which each position in the genome can be in one of three states IBD = 0, IBD = 1 or IBD = 2. The three states correspond to 0,1, or 2 pairs of chromosomes being identical by descent (Fig 1b). For efficiency and to avoid strong effects of linkage disequilibrium (LD), transitions are allowed only between ~2cM blocks, which are pre-calculated using a recombination rate map [22]. In each block, each one of the two haploid pairs of donor-recipient genomes can be in IBD or no-IBD state. The transitions between the IBD states for each haploid pair depend on the average genetic distance between the blocks and the marginal probability of the pair to be IBD, similar to the *plink* method [13]. In short, consider two haploids (*c*_1_ and *c*_2_) that share a common diploid ancestor with *c*_1_ and *c*_2_ separated by *m* ≥ 2 meiosis events. *m* = 1 –*log*_2_(*P*_*IBD*_) where *P*_*IBD*_ ∈ [0,1] is the marginal probability of the pair to be in IBD state. We define *l*_*b*,*b*+1_ to be the genetic distance between two neighboring loci *b*,*b*+1 (here, approximated by the average genetic distance between blocks in cMorgan units). The probability of an odd number of recombination events is $\theta_{b,b+1}=\;\frac{1-e^{\frac{-2*l_{b,b+1}}{100}}}{2}$. We also define *y*_1_(*θ*_*b*,*b*+1_, *m*) = (1 − *θ*_*b*,*b*+1_)^*m*−2^ and *y*_2_(*θ*_*b*,*b*+1_) = (1 − *θ*_*b*,*b*+1_)^2^ + *θ*_*b*,*b*+1_^2^. The transition matrix for two haploids is:

$$T_{b,b+1}\left(m_{i}\right)=\left[\begin{array}{cc} 1-\;\left(\frac{1-{\text{y}}_{1}\left(\theta_{b,b+1},m_{1}\right){\text{*y}}_{2}\left(\theta_{b,b+1}\right)}{2^{{\text{m}}_{i}-1}-1}\right) & \frac{1-{\text{y}}_{1}\left(\theta_{b,b+1},m_{1}\right){\text{*y}}_{2}\left(\theta_{b,b+1}\right)}{2^{{\text{m}}_{i}-1}-1}\\ 1-{\text{y}}_{1}\left(\theta_{b,b+1},m_{1}\right){\text{*y}}_{2}\left(\theta_{b,b+1}\right) & {\text{y}}_{1}\left(\theta_{b,b+1},m_{1}\right){\text{*y}}_{2}\left(\theta_{b,b+1}\right) \end{array}\right]$$

where *i* ∈ {1,2}. The transition matrix for the IBD states of the two pairs of haploids is a simple combination of the two haploid pairs transition matrices and depends on their two IBD parameters: $P_{IBD}^{I}$ and $P_{IBD}^{II}$. Similar to PLINK, we limit $P_{IBD}^{I}$ and $P_{IBD}^{II}$ to be at most 0.5. This excludes parent-child relations from the donor-recipient relationships. Although we did not address it in this work, dd-cfDNA of parent-child donor-recipient can be estimated by multiply by two the percent dd-cfDNA predicted when restricting the donor and recipient to be unrelated.

The emissions probabilities of each SNP in each IBD state are similar to the likelihood function above with one difference—the probability of the donor genotype depends also on the recipient genotype (in addition to its dependence on the ancestral population):

$$P_{i}\left(\left(R_{i_{1}}^{*},R_{i_{2}}^{*}\right),\;C_{i_{1}}^{*},\;\ldots \;C_{i_{K_{i}}}^{*}|d,e_{s},e_{g,}pop_{m}\right)=\prod_{j=1}^{K_{i}}\sum_{c,r_{1},r_{1},d_{1},d_{2,c}\in \left\{A,B\right\}}\left(\begin{array}{c} P\left(C_{i_{j}}^{*}|C_{i_{j}}=c;s_{e}\right)\\ {}*P\left(C_{i_{j}}\text{|}(R_{i_{1}},R_{i_{2}}\text{)},(D_{i_{1}},D_{i_{2}}\right);d)\\ {}*P\left(D_{i_{1}}=d_{1},D_{i_{2}}=d_{2}|R_{i_{1}}=r_{1},R_{i_{2}}=r_{2},IBD_{i};pop_{m}\right)\\ {}*P\left(R_{i_{1}}=r_{1},R_{i_{2}}=r_{2}\right)\\ {}*P\left(R_{i_{1}}^{*},R_{i_{2}}^{*}|R_{i_{1}}=r_{1},R_{i_{2}}=r_{2};g_{e}\right) \end{array}\right)$$

Tables 1–3 show $P\left(D_{i_{1}}=d_{1},D_{i_{2}}=d_{2}|R_{i_{1}}=r_{1},R_{i_{2}}=r_{2},IBD_{i};pop_{m}\right)$ for a bi-allelic SNP_i,_ which has two possible alleles: A and B that are occur with frequency *f*_*A*_ and *f*_*B*_ in *Pop*_*m*_ respectively, for *IBD*_*i*_ = 0,1,2.

**Table 1:** Probabilities of donor genotypes of a bi-allelic SNP conditioning on recipient genotype, donor population and no IBD. The table shows $P\left(D_{i_{1}}=d_{1},D_{i_{2}}=d_{2}|R_{i_{1}}=r_{1},R_{i_{2}}=r_{2},IBD_{i}=0;pop_{m}\right)$ for a bi-allelic SNP_i,_ which has two possible alleles: A and B that are occur with frequency *f*_*A*_ and *f*_*B*_ in *Pop*_*m*_ respectively.

	Donor genotype			
Conditioning on the recipient genoptype		AA	AB or BA	BB
	AA	$f_{A}^{2}$	2*f*_*A*_*f*_*B*_	$f_{B}^{2}$
	AB or BA	$f_{A}^{2}$	2*f*_*A*_*f*_*B*_	$f_{B}^{2}$
	BB	$f_{A}^{2}$	2*f*_*A*_*f*_*B*_	$f_{B}^{2}$

**Table 2:** Probabilities of donor genotypes of a bi-allelic SNP conditioning on recipient genotype, donor population and IBD between one haploid pair of donor-recipient genomes. The table shows $P\left(D_{i_{1}}=d_{1},D_{i_{2}}=d_{2}|R_{i_{1}}=r_{1},R_{i_{2}}=r_{2},IBD_{i}=1;pop_{m}\right)$ for a bi-allelic SNP_i,_ which has two possible alleles: A and B that are occur with frequency *f*_*A*_ and *f*_*B*_ in *Pop*_*m*_ respectively.

	Donor genotype			
Conditioning on the recipient genoptype		AA	AB or BA	BB
	AA	*f*_*A*_	*f*_*B*_	0
	AB or BA	0.5*f*_*A*_	(0.5*f*_*A*_ + 0.5*f*_*B*_)	0.5*f*_*B*_
	BB	0	*f*_*A*_	*f*_*B*_

**Table 3:** Probabilities of donor genotypes of a bi-allelic SNP conditioning on recipient genotype, donor population and IBD between two haploid pairs of donor-recipient genomes. The table shows $P\left(D_{i_{1}}=d_{1},D_{i_{2}}=d_{2}|R_{i_{1}}=r_{1},R_{i_{2}}=r_{2},IBD_{i}=2;pop_{m}\right)$ for a bi-allelic SNP_i,_ which has two possible alleles: A and B that are occur with frequency *f*_*A*_ and *f*_*B*_ in *Pop*_*m*_ respectively.

	Donor genotype			
Conditioning on the recipient genoptype		AA	AB or BA	BB
	AA	1	0	0
	AB or BA	0	1	0
	BB	0	0	1

Putting it together, the parameters of interest of the model is *d*—the fraction of dd-cfDNA, and the nuisance parameters are *e*_*s*_—sequencing error probability, *e*_*g*_—genotyping probability, *pop*_*m*_—donor's ancestral population and $P_{IBD}^{I}$ and $P_{IBD}^{II}$ IBD probabilities for two haploid pairs. We used the forward algorithm to integrate the likelihood over all possible HMM paths for specific parameter values, and optimize the likelihood using L-BFGS-B.

### Comparing one-genome and two-genomes methods predictability of organ rejection

To assess how well each method dd-cfDNA predictions can be used to discriminate between different levels of heart and lung rejection, we computed the area under the curve (AUC) of the receiver operating characteristic (ROC) similar to how this was done in our previous publications [8,9]: the dd-cfDNA prediction of one lung donation were doubled to match the levels of two lungs donations and measurements previous to 14 and 60 days following heart and lung transplant correspondingly were removed from the analysis. A two-sided DeLong test [31] (Implemented in R pROC package [32]) was used to assess the significance of the difference between two corresponding ROC curves.

### Simulating organ transplant recipient samples

To simulate organ transplant recipient samples, we used the pre-transplant samples from the bone marrow cohort of patients I2, I3, and I8. In each simulation, we considered one sample as the recipient and one sample as the donor. We merged the data from the two samples and filtered SNPs that were clear genotyping errors (0.2% of the SNPs; homozygote SNPs with cfDNA reads that contain the non-present allele). We then mixed randomly sampled reads from each of the two samples at a specific ratio. We considered the fraction of the second sample as the simulated fraction of dd-cfDNA. To simulate IBD we used the same HMM transition matrix as used for the inference. In each transition between genetics blocks, we randomly selected one of the states for the next block using the transition probabilities matrix. If a state has an IBD state of 1, for each “donor” read we randomly selected with probability 0.5 read from the first sample and with probability 0.5 read from the second sample. In IBD state 2 all the “donor” reads were selected from the first sample and in IBD state 0 all “donor” reads were from the second sample. We down sampled the SNPs by taking K SNPs in each genetic block with the highest alternative allele frequency in 1000 genomes project (we simulated K = 100,50 and 20). We randomly down sampled the reads to a desired average coverage (we simulated average coverage of 1.75, 1, 0.5. 0.33 and 0.1). To run many simulations, we saved running time by limiting the inference to a single population. We used the population that was inferred for the donor, since for these cases (I2, I3 and I8) the donor was a sibling of the recipient. We show in S12 Fig that this gives similar results to using all the populations for a subset of the simulations.

## Supporting information

S1 Table Lung transplant cohort. Clinical and experimental information and predicted dd-cfDNA levels using two- and one-genome methods of samples drawn from lung transplant recipients.
(XLSX)

S2 Table Heart transplant cohort. Clinical and experimental information and predicted dd-cfDNA levels using two- and one-genome methods of samples drawn from heart transplant recipients.
(XLSX)

S3 Table Bone marrow transplant cohort. Experimental information and predicted dd-cfDNA levels using two- and one-genome methods of samples drawn from bone marrow transplant recipients.
(XLSX)

S4 Table Clinical events in bone marrow transplant recipients. Engraftment, GVHD, repalse and VOD diagnosis dates of marrow transplant recipients.
(XLSX)

S5 Table Results of simulation. Method performances in various simulated levels of dd-cfDNA, genotyped SNPs and cfDNA sequencing depth.
(XLSX)

S6 Table Results of simulations comparing multi and single population optimization. Comparing predictions of percent of dd-cfDNA using all 1000 genomes populations and a single population that was inferred for the simulated donor sibling in the real data.
(XLSX)

S1 Fig cfDNA sequencing and genotyping data processing pipeline. Illustration of the pipeline used to retrieve allele counts in cfDNA fragments for each recipient-genotyped SNP from the raw cfDNA sequencing and genotyping measurements.
(PDF)

S2 Fig Differences between estimation of dd-cfDNA levels by one- and two-genomes methods. Assuming the two-genomes model is the gold standard, we assess the absolute error of the one-genome method in lung (a) heart (b) and bone marrow (c) recipients. Note that this may be an over estimated error, since the two-genomes method is probably not completely accurate.
(PDF)

S3 Fig Comparison of organ rejection states using one-genome method and two-genomes method with error correction in heart and lung transplant recipients. (a) and (b) show a comparison of one- and two-genomes methods predictability of organ rejection. In opposed to Fig 2, here the two-genome prediction was corrected by error estimation. Each bar shows the area under the curve (AUC) of discriminating between two rejection states as measured using biopsies using dd-cfDNA fraction estimates [8,9]. Error bars marks AUC 95% confidence interval. The significance of the difference between corresponding receiver operating characteristic (ROC) of the one-genome and two-genomes was done using DeLong two sided test [32,31].
(PDF)

S4 Fig A comparison between predicted levels of dd-cfDNA and the fraction of reads that map to the X chromosome when recipient and donor sex are different. Patients I1, I2 and I4 the recipient are males with female donors; patient I8 is a female with a male donor.
(PDF)

S5 Fig A comparison between one- and two-genomes methods predictions of cfDNA levels in bone marrow transplant patients. Each panel shows results for a single patient. Dotted-dash line marks day in which engraftment was detected (absolute neutrophil count (ANC) > 500 for three consecutive days). Purple dashed lines mark clinical diagnoses. Notice that the predictions of one-genome method that learns IBD are similar to the prediction of the two-genomes method, while fixing the one-genome method to non-related recipient and donor state (IBD = 0) underestimate the dd-cfDNA fraction.
(PDF)

S6 Fig Method performance for different levels of relatedness of recipient and donor. A comparison of the predicted and simulated levels of dd-cfDNA (a) and the corresponding absolute error (b) for different levels of relatedness of the donor and the recipient. dd-cfDNA levels were estimated using 640-700K SNPs and cfDNA sequencing depth of 1.76–1.78x (corresponding to 26M 100bp paired ends reads or 210 USD in our academic sequencing center). Larger markers the median result for a simulated dd-cfDNA level, smaller, unconnected markers show independent simulations. Lines show spline interpolation of the median values.
(PDF)

S7 Fig Method performance for different levels of relatedness of recipient and donor with 1x cfDNA sequencing coverage input. A comparison of the predicted and simulated levels of dd-cfDNA (a) and the corresponding absolute error (b) for different levels of relatedness of the donor and the recipient. dd-cfDNA levels were estimated using 640-700K SNPs and cfDNA sequencing depth of 1x (corresponding to 15M 100bp paired ends reads or 120 USD in our academic sequencing center). Larger markers the median result for a simulated dd-cfDNA level, smaller, unconnected markers show independent simulations. Lines show spline interpolation of the median values.
(PDF)

S8 Fig Method performance for different levels of relatedness of recipient and donor with 150K SNPs and 1x cfDNA sequencing coverage input. A comparison of the predicted and simulated levels of dd-cfDNA (a) and the corresponding absolute error (b) for different levels of relatedness of the donor and the recipient. dd-cfDNA levels were estimated using 157K SNPs and cfDNA sequencing depth of 1x (corresponding to 15M 100bp paired ends reads or 120 USD in our academic sequencing center). Larger markers the median result for a simulated dd-cfDNA level, smaller, unconnected markers show independent simulations. Lines show spline interpolation of the median values.
(PDF)

S9 Fig Method performance for different levels of relatedness of recipient and donor with <1x cfDNA sequencing coverage input. A comparison of the predicted and simulated levels of dd-cfDNA (a,c) and the corresponding absolute error (b,d) for different levels of relatedness of the donor and the recipient. dd-cfDNA levels were estimated using 640-700K SNPs and cfDNA sequencing depth of 0.5x (a,b) or 0.33x (c,d). Larger markers the median result for a simulated dd-cfDNA level, smaller, unconnected markers show independent simulations. Lines show spline interpolation of the median values.
(PDF)

S10 Fig Method performance for unrelated recipient and donor using different number of cfDNA sequencing reads. A comparison of the predicted and simulated levels of dd-cfDNA (a) and the corresponding absolute error (b) for different levels of relatedness of the donor and the recipient. dd-cfDNA levels were estimated using 640-700K SNPs and cfDNA sequencing depth of 0.1–1.78x. Larger markers the median result for a simulated dd-cfDNA level, smaller, unconnected markers show independent simulations. Lines show spline interpolation of the median values.
(PDF)

S11 Fig Method performance for unrelated recipient and donor using different number of genotyped SNPs. A comparison of the predicted and simulated levels of dd-cfDNA (a) and the corresponding absolute error (b) for different levels of relatedness of the donor and the recipient. dd-cfDNA levels were estimated using 640-700K SNPs and cfDNA sequencing depth of 0.1–1.78x. Larger markers the median result for a simulated dd-cfDNA level, smaller, unconnected marker show independent simulations. Lines show spline interpolation of the median values.
(PDF)

S12 Fig Comparing one-genome method predicted percent of cfDNA when optimizing over all 1000 genome populations to when optimizing over a single population, over simulated data. Comparing predicted percent cf-ddDNA using all 1000 genomes project populations (x-axis) to using the single population (y-axis) over 93 simulations (Pearson’s R^2^ = 0.995). The single population was the population that was inferred for the simulated “donor” sibling in real samples.
(PDF)
