# Supplementary material for: Quantification of transplant-derived circulating cell-free DNA in absence of a donor genotype
Source: PLoS Comput Biol. 2017 Aug 3;13(8):e1005629. doi: 10.1371/journal.pcbi.1005629 (PMC5542400; doi:10.1371/journal.pcbi.1005629)
Supplement: S1 Fig — Illustration of the pipeline used to retrieve allele counts in cfDNA fragments for each recipient-genotyped SNP from the raw cfDNA sequencing and genotyping measurements. (PDF) [file pcbi.1005629.s007.pdf]

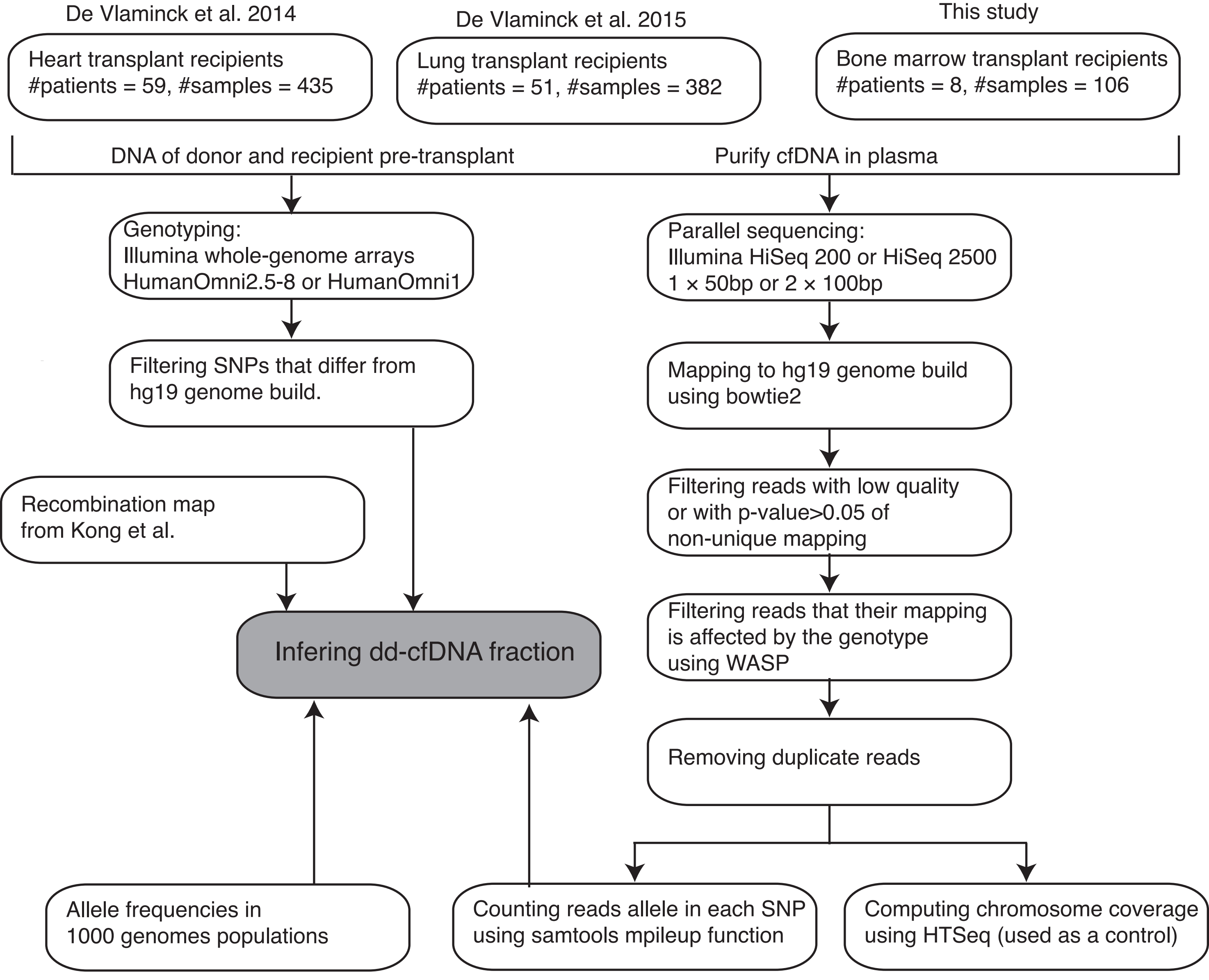
De Vlaminck et al. 2014
Heart transplant recipients
#patients = 59, #samples = 435
De Vlaminck et al. 2015
Lung transplant recipients
#patients = 51, #samples = 382
This study
Bone marrow transplant recipients
#patients = 8, #samples = 106
DNA of donor and recipient pre-transplant
Purify cfDNA in plasma
Genotyping:
Illumina whole-genome arrays
HumanOmni2.5-8 or HumanOmni1
Parallel sequencing:
Illumina HiSeq 200 or HiSeq 2500
1 × 50bp or 2 × 100bp
Filtering SNPs that differ from
hg19 genome build.
Mapping to hg19 genome build
using bowtie2
Recombination map
from Kong et al.
Filtering reads with low quality
or with p-value>0.05 of
non-unique mapping
Infering dd-cfDNA fraction
Filtering reads that their mapping
is affected by the genotype
using WASP
Removing duplicate reads
Allele frequencies in
1000 genomes populations
Counting reads allele in each SNP
using samtools mpileup function
Computing chromosome coverage
using HTSeq (used as a control)
