# Supplementary figures and images for: Quantification of transplant-derived circulating cell-free DNA in absence of a donor genotype
Source: PLoS Comput Biol. 2017 Aug 3;13(8):e1005629. doi: 10.1371/journal.pcbi.1005629 (PMC5542400; doi:10.1371/journal.pcbi.1005629)

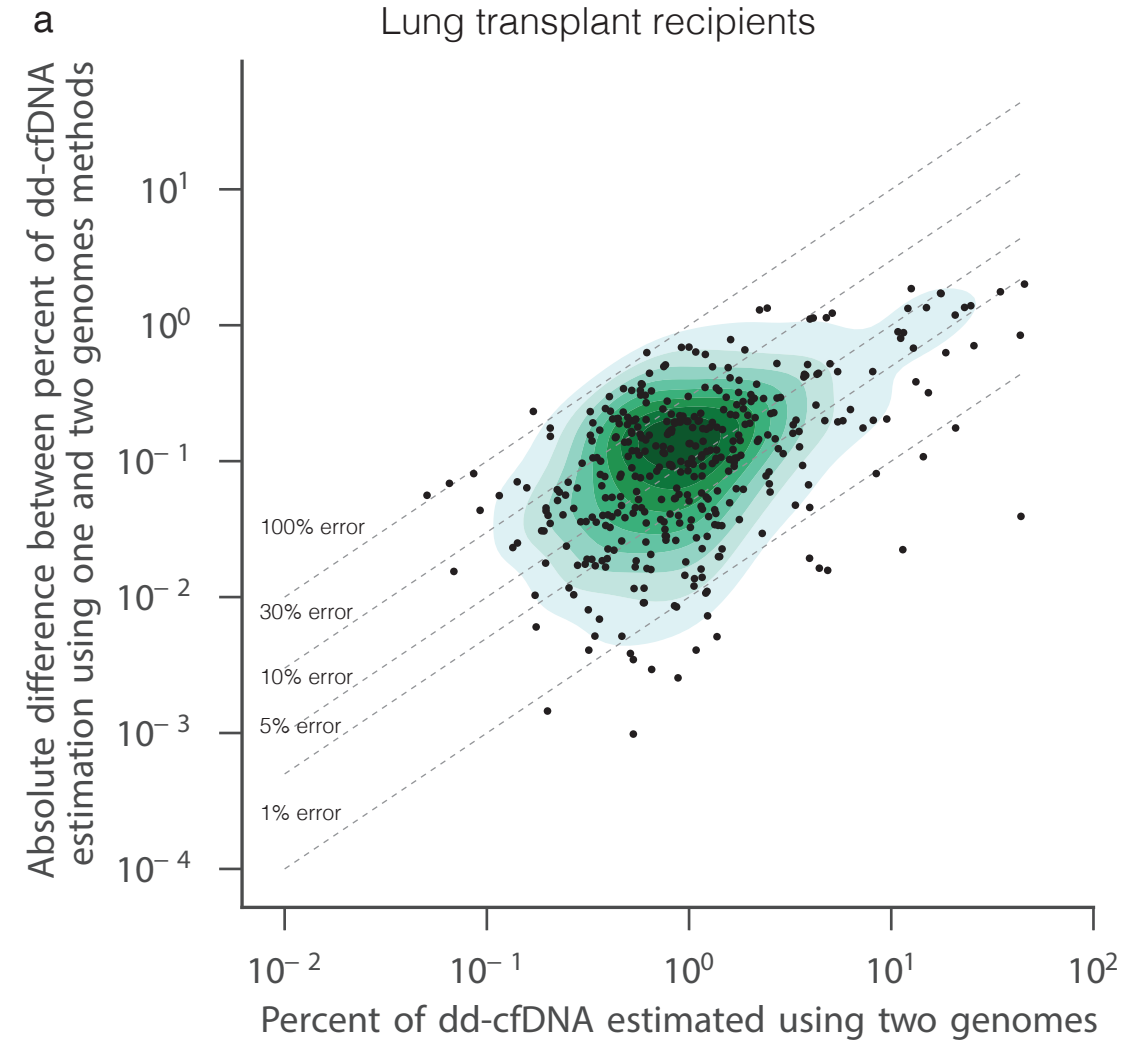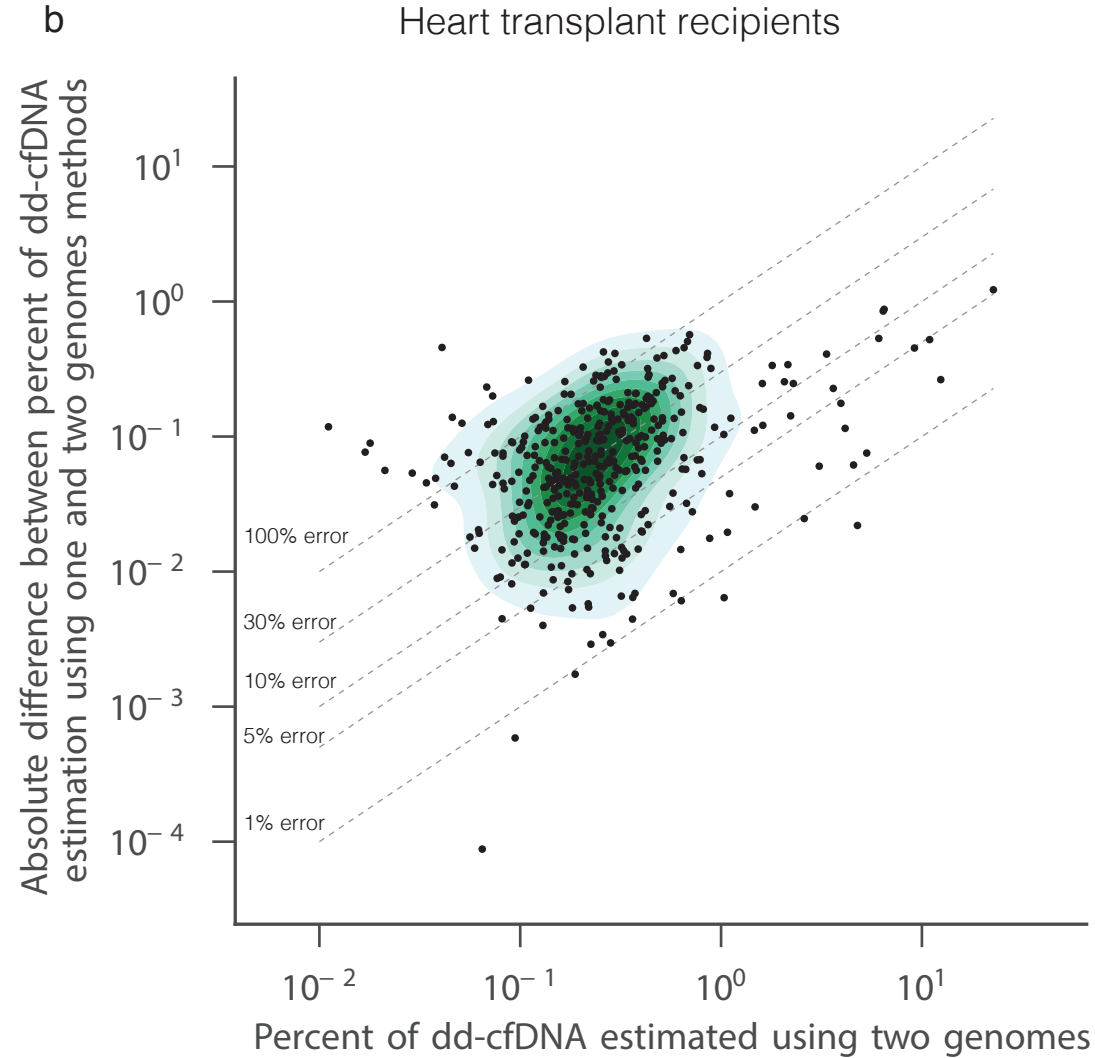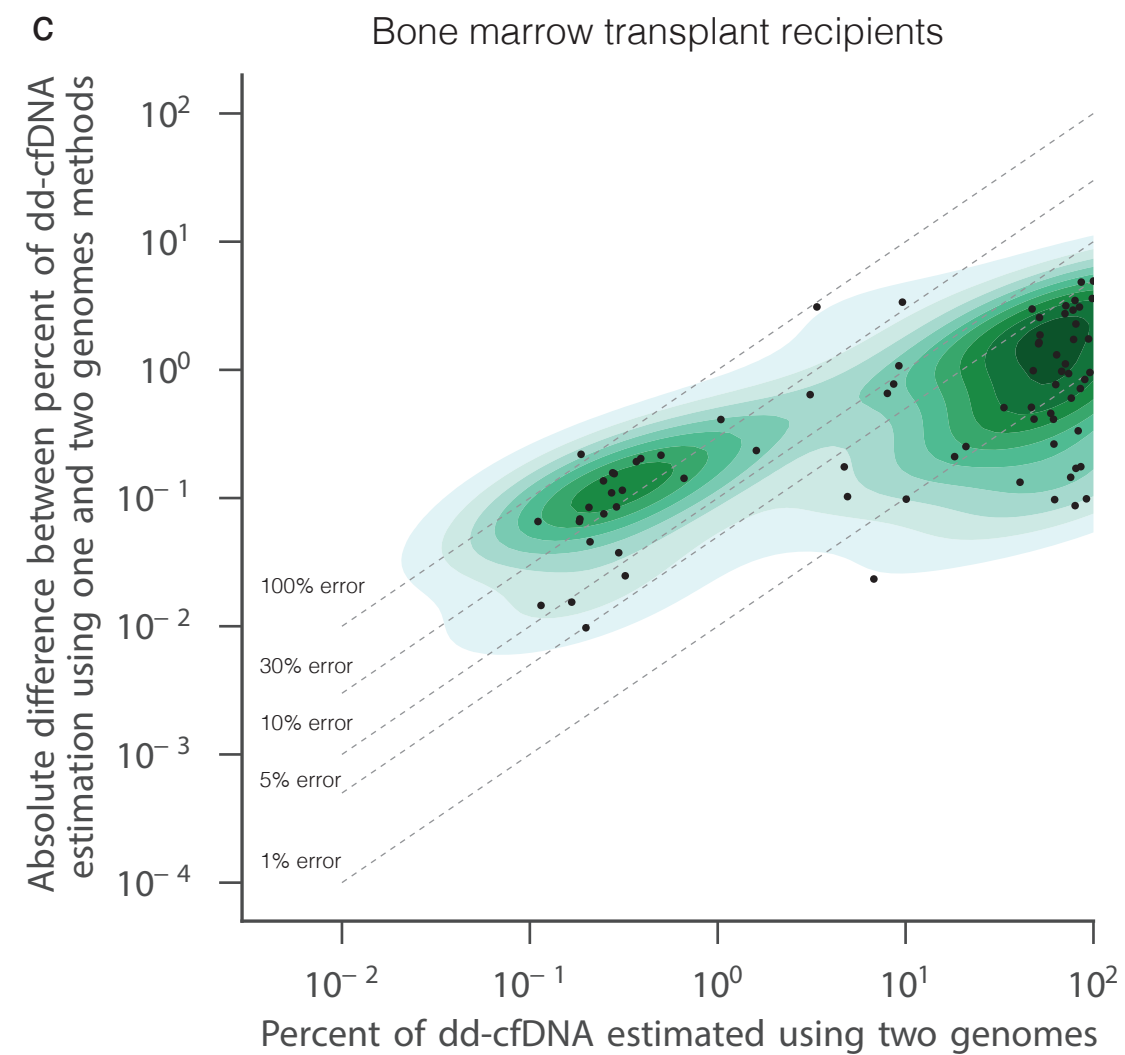

Supplement: S2 Fig — Assuming the two-genomes model is the gold standard, we assess the absolute error of the one-genome method in lung (a) heart (b) and bone marrow (c) recipients. Note that this may be an over estimated error, since the two-genomes method is probably not completely accurate. (PDF) [file pcbi.1005629.s008.pdf]

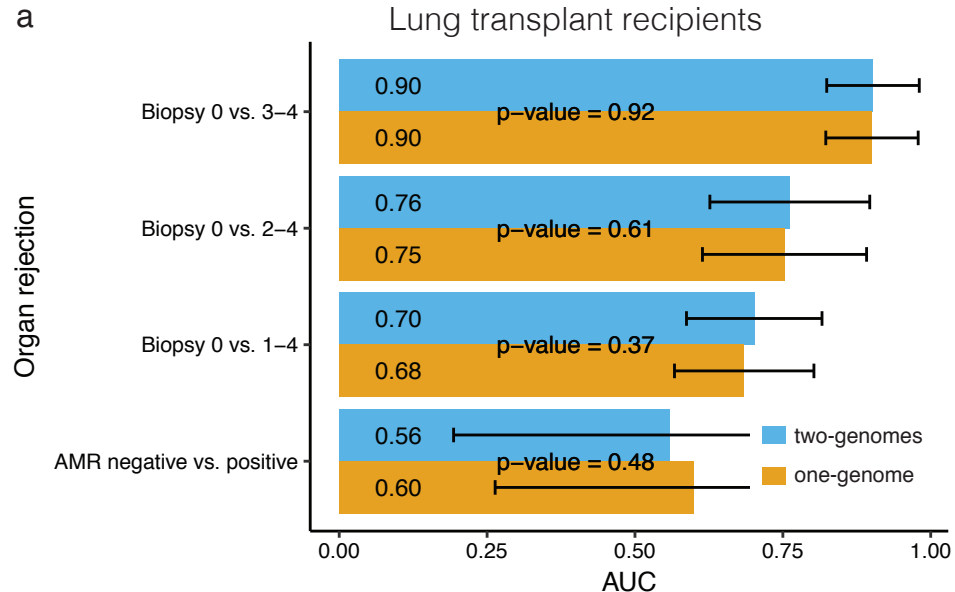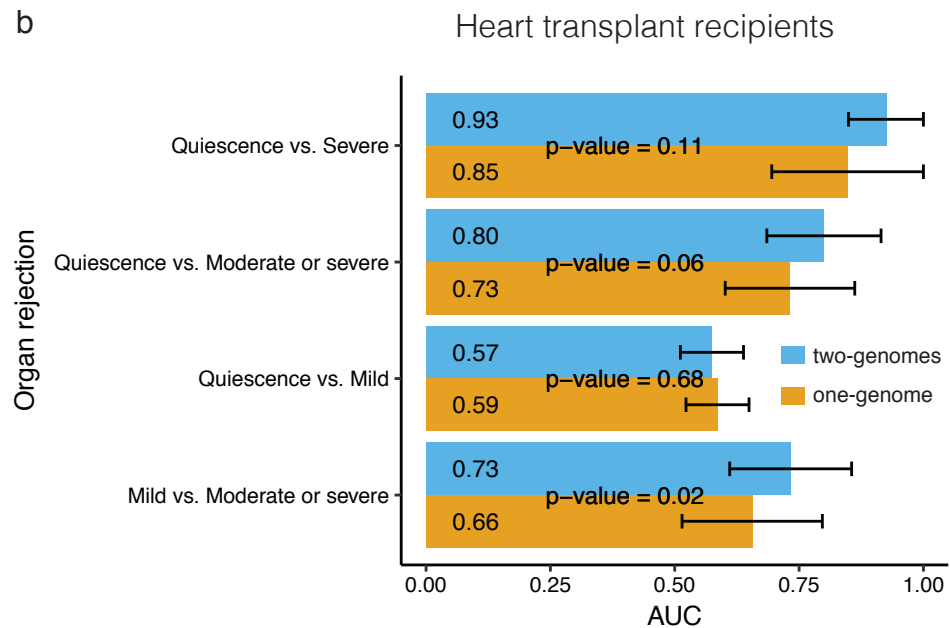

Supplement: S3 Fig — (a) and (b) show a comparison of one- and two-genomes methods predictability of organ rejection. In opposed to Fig 2, here the two-genome prediction was corrected by error estimation. Each bar shows the area under the curve (AUC) of discriminating between two rejection states as measured using biopsies using dd-cfDNA fraction estimates [8,9]. Error bars marks AUC 95% confidence interval. The significance of the difference between corresponding receiver operating characteristic (ROC) of the one-genome and two-genomes was done using DeLong two sided test [32,31]. (PDF) [file pcbi.1005629.s009.pdf]

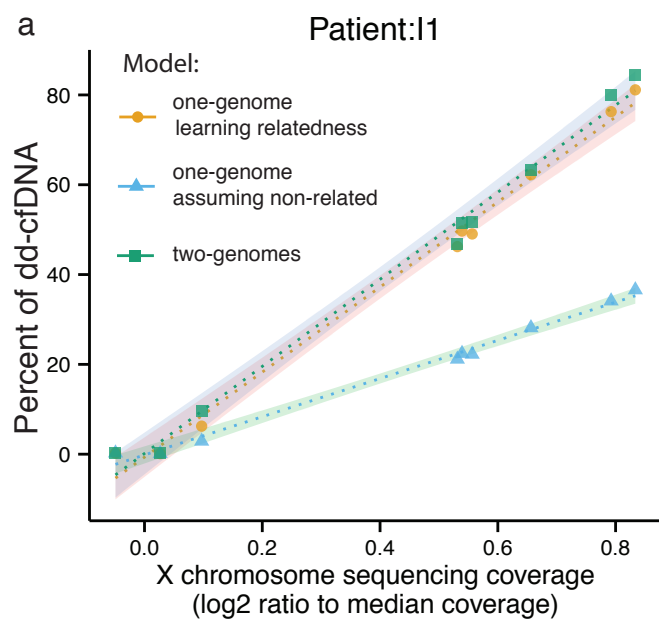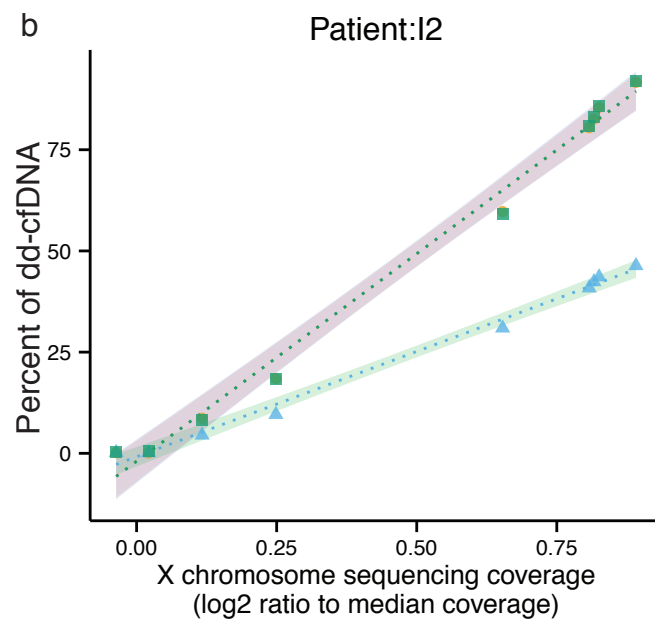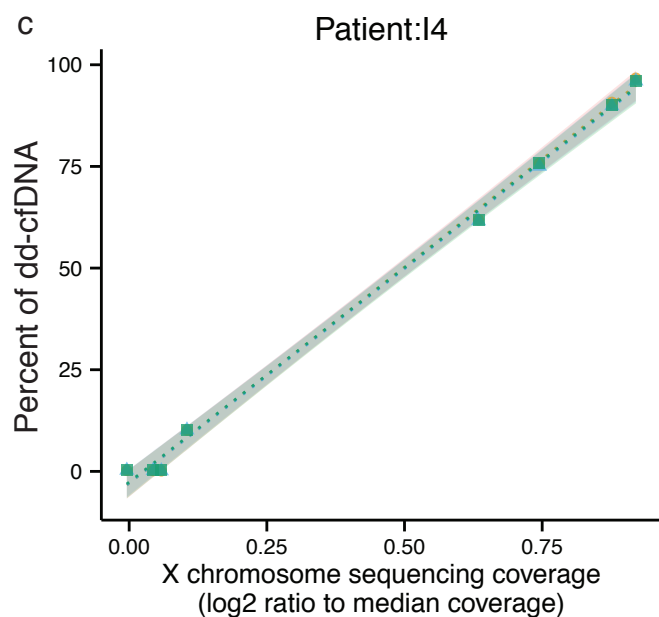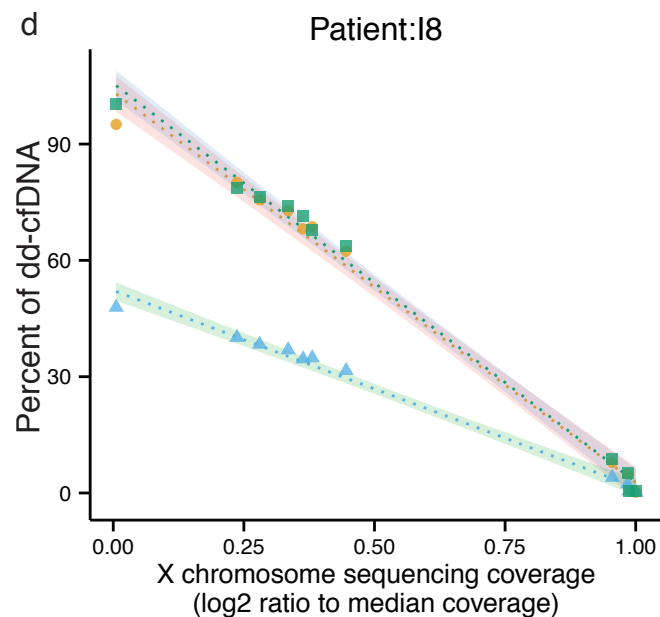

Supplement: S4 Fig — Patients I1, I2 and I4 the recipient are males with female donors; patient I8 is a female with a male donor. (PDF) [file pcbi.1005629.s010.pdf]

Model comparison of predictions of percent of dd-cfDNA in bone marrow transplant recipients

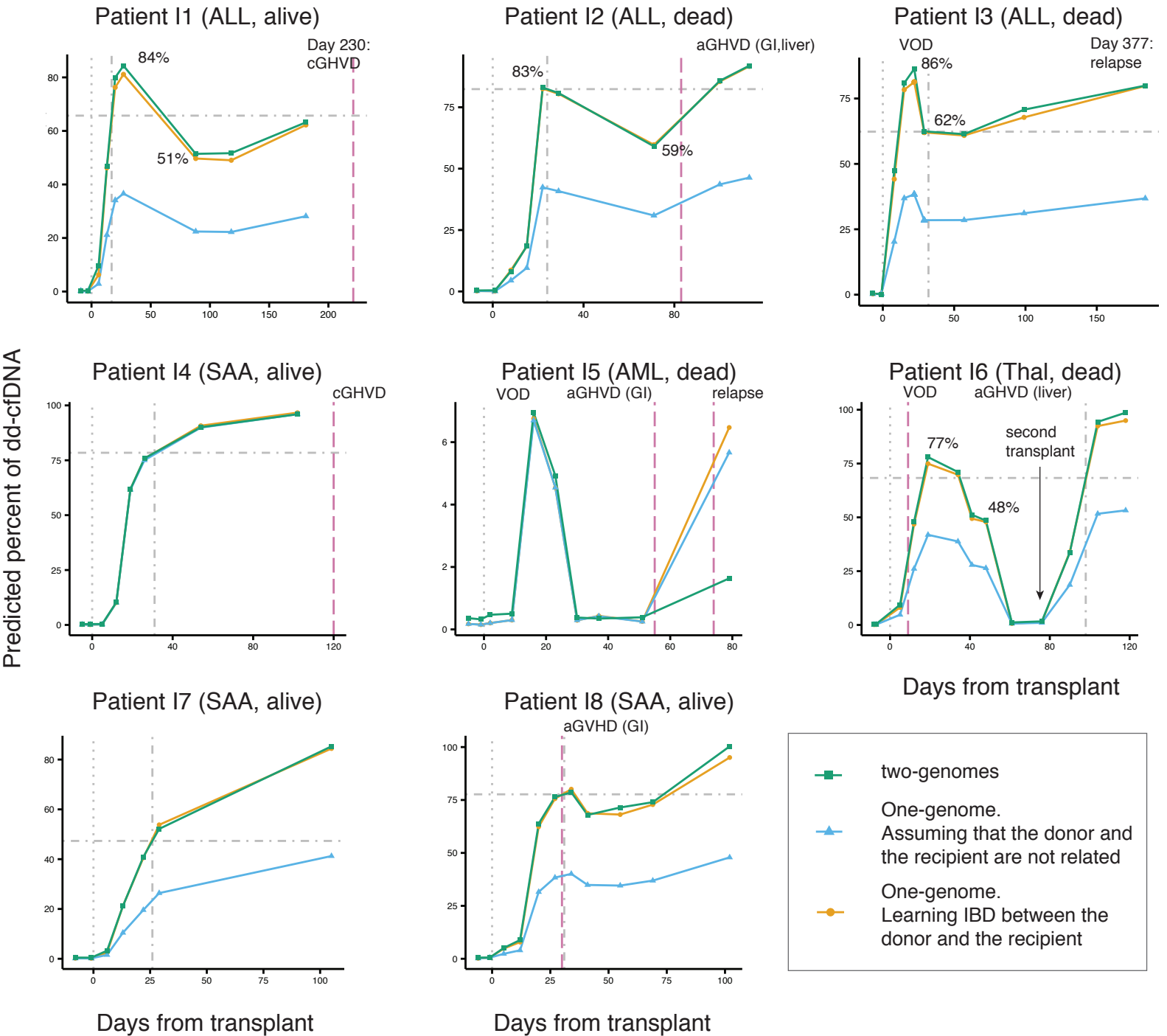

Supplement: S5 Fig — Each panel shows results for a single patient. Dotted-dash line marks day in which engraftment was detected (absolute neutrophil count (ANC) > 500 for three consecutive days). Purple dashed lines mark clinical diagnoses. Notice that the predictions of one-genome method that learns IBD are similar to the prediction of the two-genomes method, while fixing the one-genome method to non-related recipient and donor state (IBD = 0) underestimate the dd-cfDNA fraction. (PDF) [file pcbi.1005629.s011.pdf]

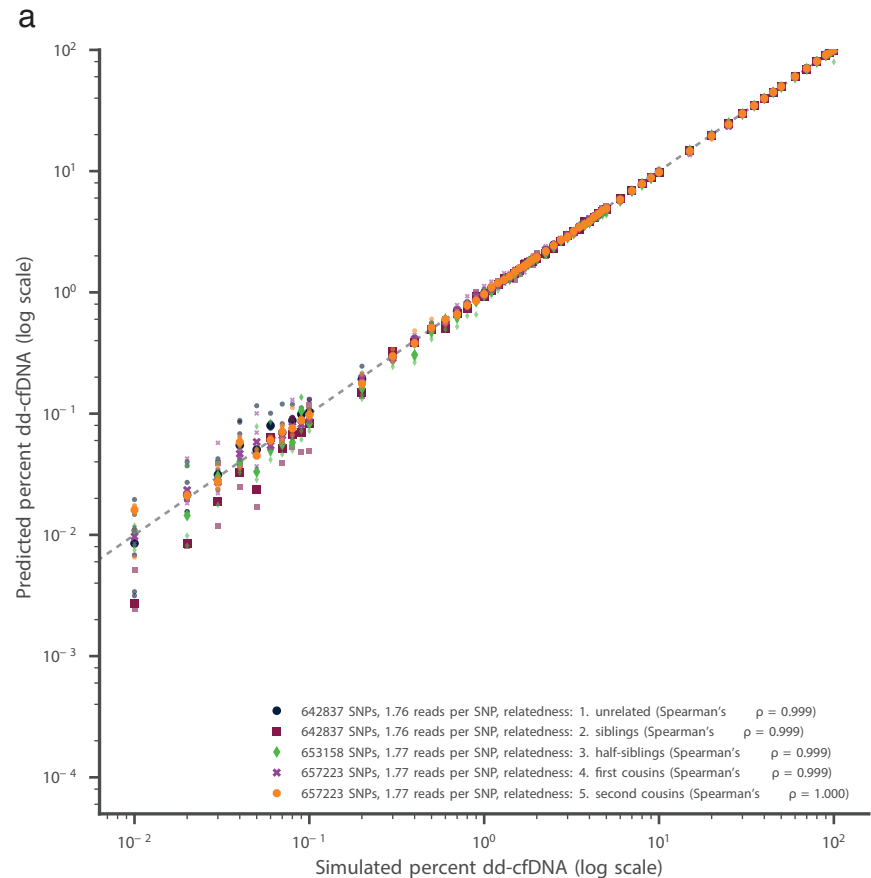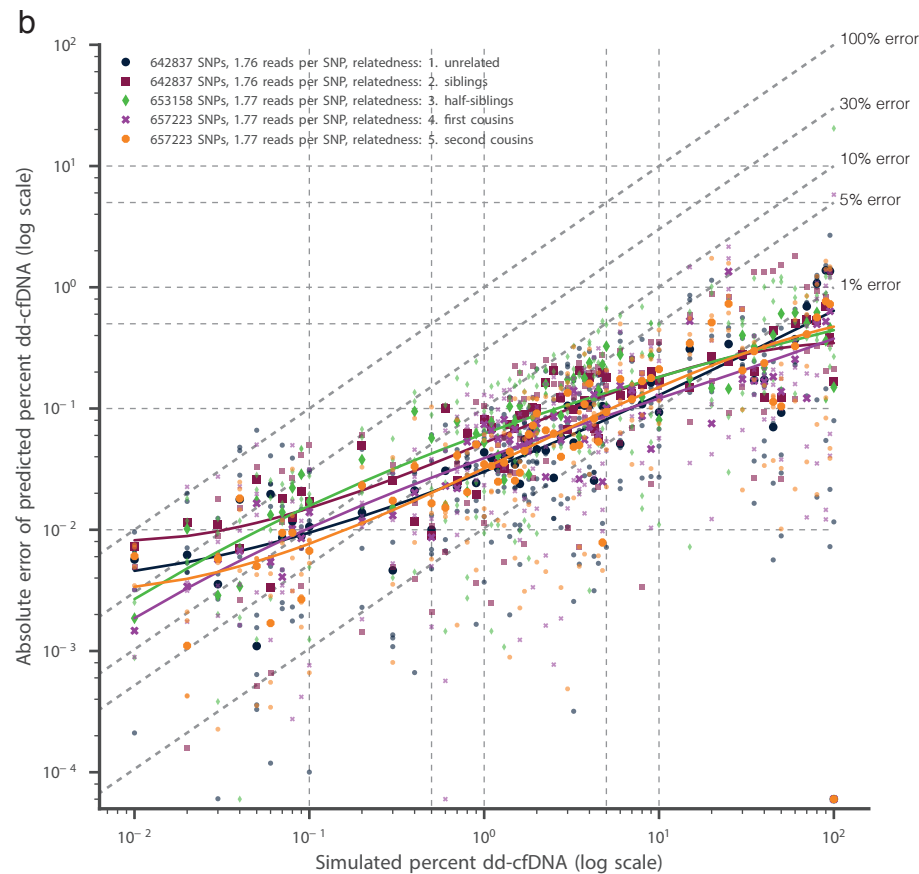

Supplement: S6 Fig — A comparison of the predicted and simulated levels of dd-cfDNA (a) and the corresponding absolute error (b) for different levels of relatedness of the donor and the recipient. dd-cfDNA levels were estimated using 640-700K SNPs and cfDNA sequencing depth of 1.76–1.78x (corresponding to 26M 100bp paired ends reads or 210 USD in our academic sequencing center). Larger markers the median result for a simulated dd-cfDNA level, smaller, unconnected markers show independent simulations. Lines show spline interpolation of the median values. (PDF) [file pcbi.1005629.s012.pdf]

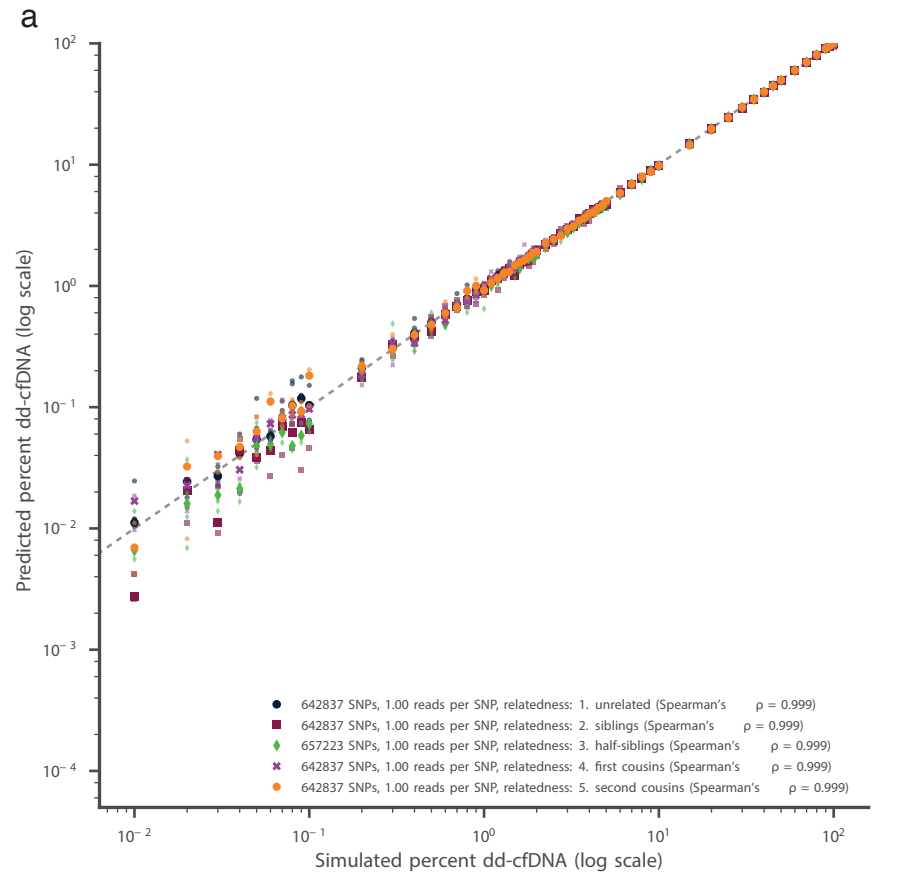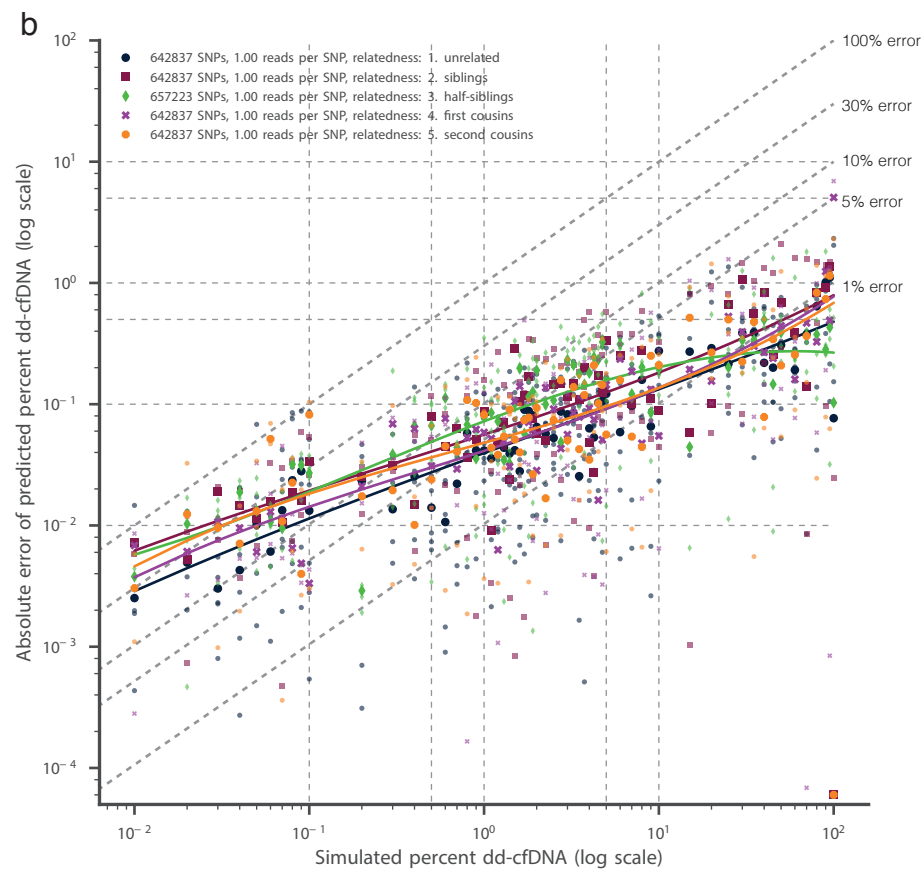

Supplement: S7 Fig — A comparison of the predicted and simulated levels of dd-cfDNA (a) and the corresponding absolute error (b) for different levels of relatedness of the donor and the recipient. dd-cfDNA levels were estimated using 640-700K SNPs and cfDNA sequencing depth of 1x (corresponding to 15M 100bp paired ends reads or 120 USD in our academic sequencing center). Larger markers the median result for a simulated dd-cfDNA level, smaller, unconnected markers show independent simulations. Lines show spline interpolation of the median values. (PDF) [file pcbi.1005629.s013.pdf]

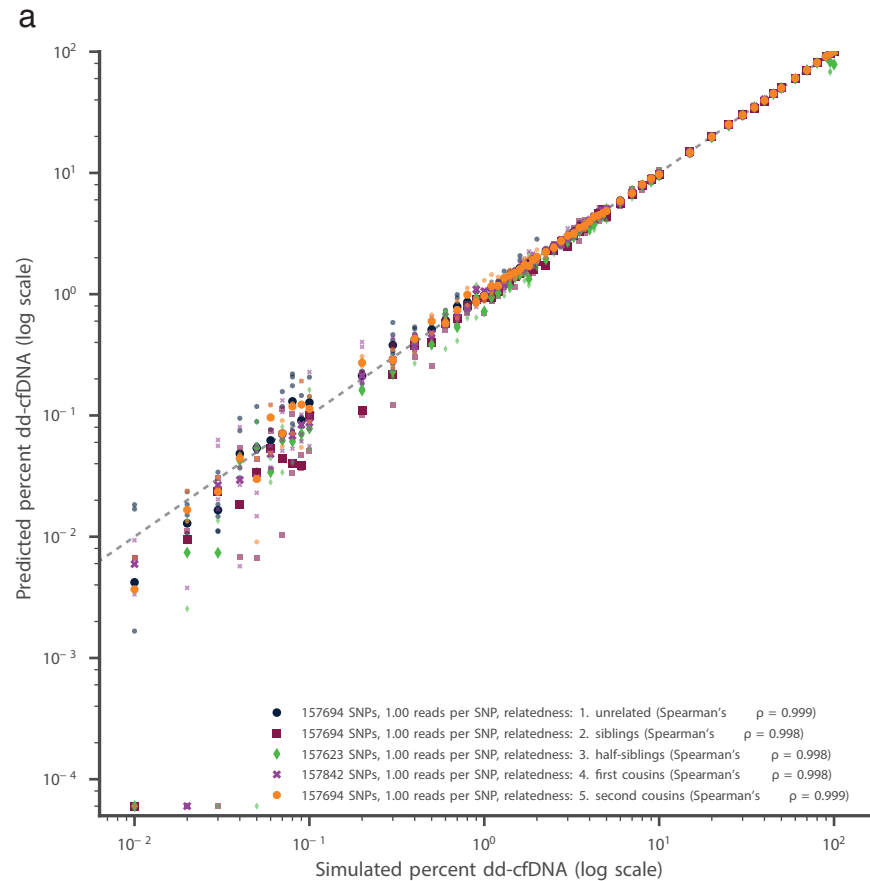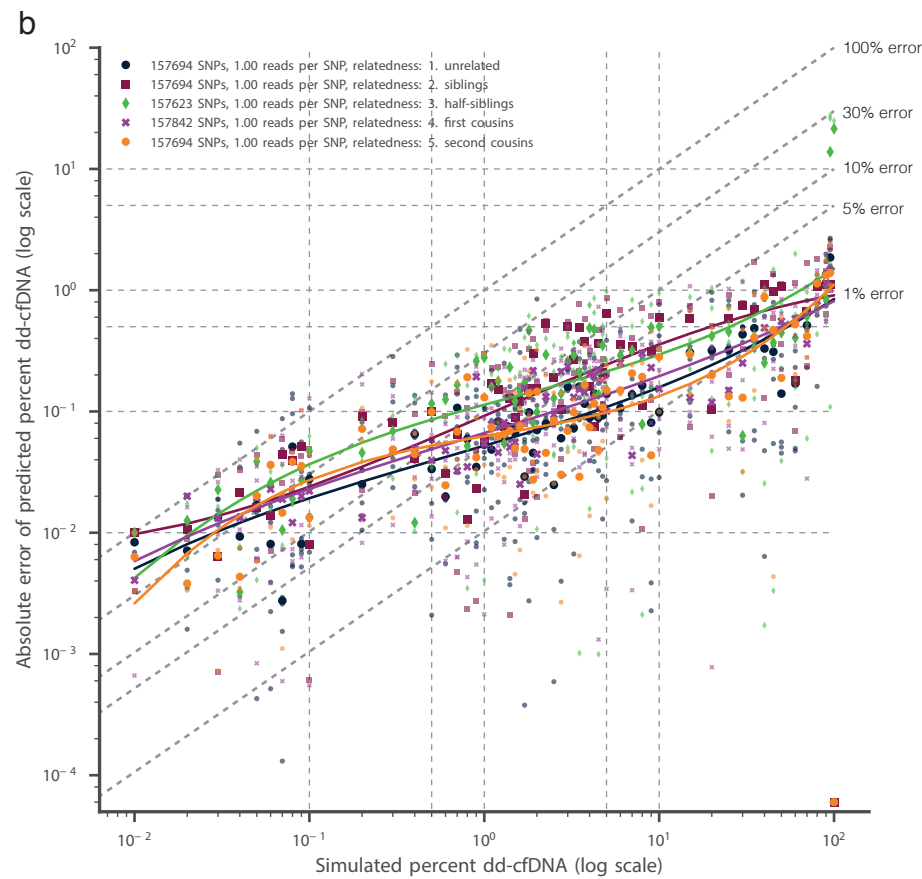

Supplement: S8 Fig — A comparison of the predicted and simulated levels of dd-cfDNA (a) and the corresponding absolute error (b) for different levels of relatedness of the donor and the recipient. dd-cfDNA levels were estimated using 157K SNPs and cfDNA sequencing depth of 1x (corresponding to 15M 100bp paired ends reads or 120 USD in our academic sequencing center). Larger markers the median result for a simulated dd-cfDNA level, smaller, unconnected markers show independent simulations. Lines show spline interpolation of the median values. (PDF) [file pcbi.1005629.s014.pdf]

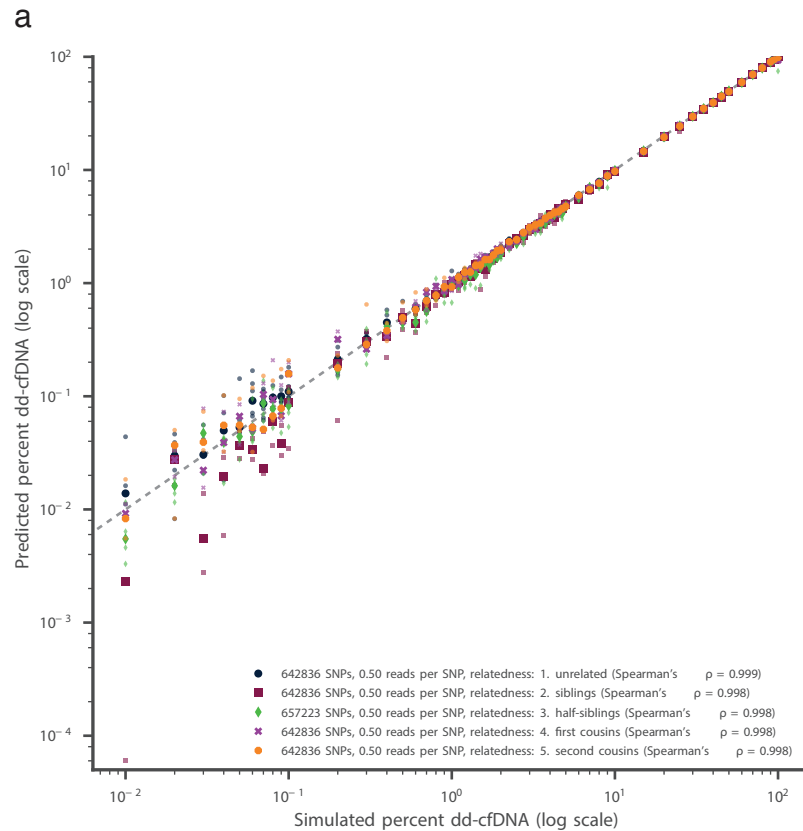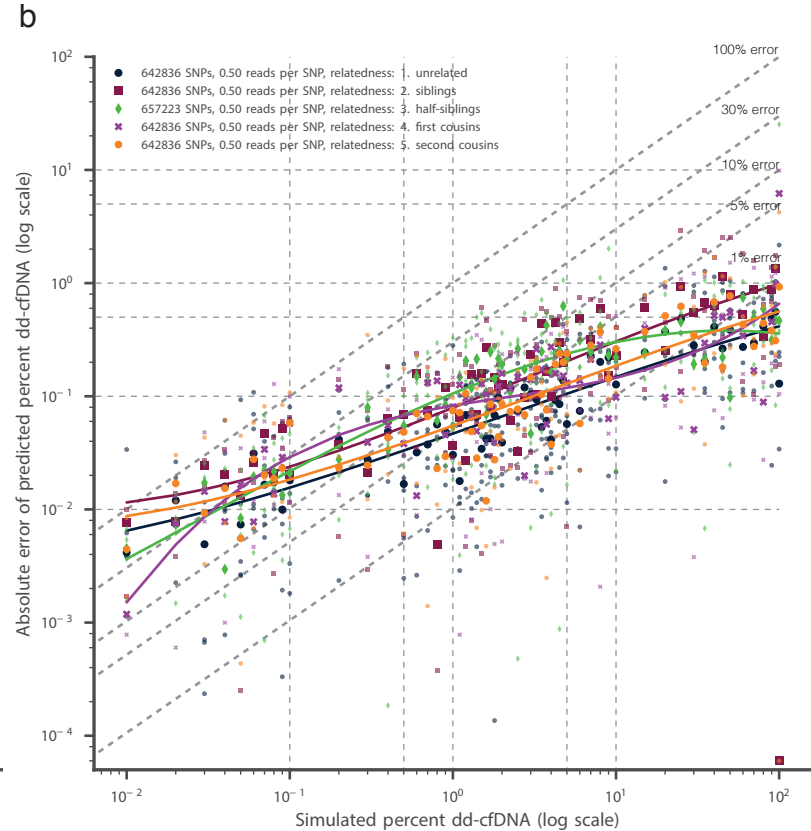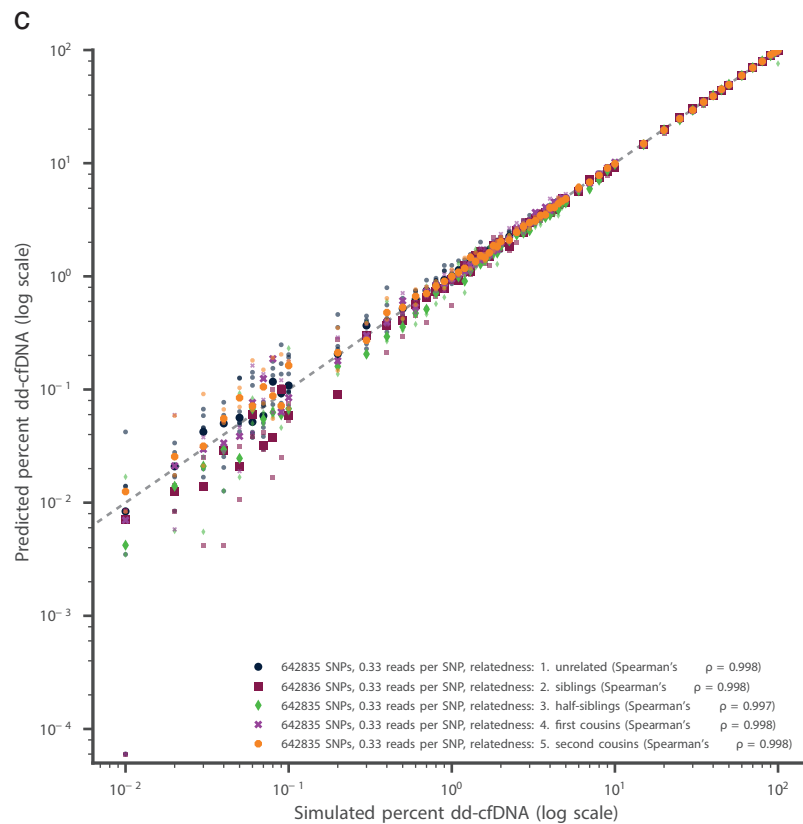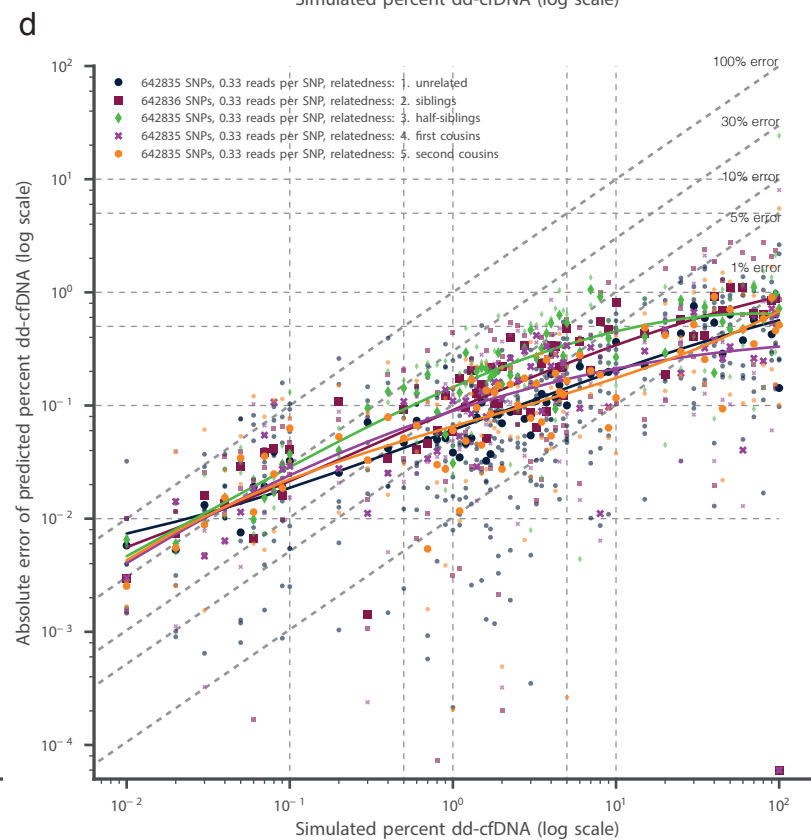

Supplement: S9 Fig — A comparison of the predicted and simulated levels of dd-cfDNA (a,c) and the corresponding absolute error (b,d) for different levels of relatedness of the donor and the recipient. dd-cfDNA levels were estimated using 640-700K SNPs and cfDNA sequencing depth of 0.5x (a,b) or 0.33x (c,d). Larger markers the median result for a simulated dd-cfDNA level, smaller, unconnected markers show independent simulations. Lines show spline interpolation of the median values. (PDF) [file pcbi.1005629.s015.pdf]

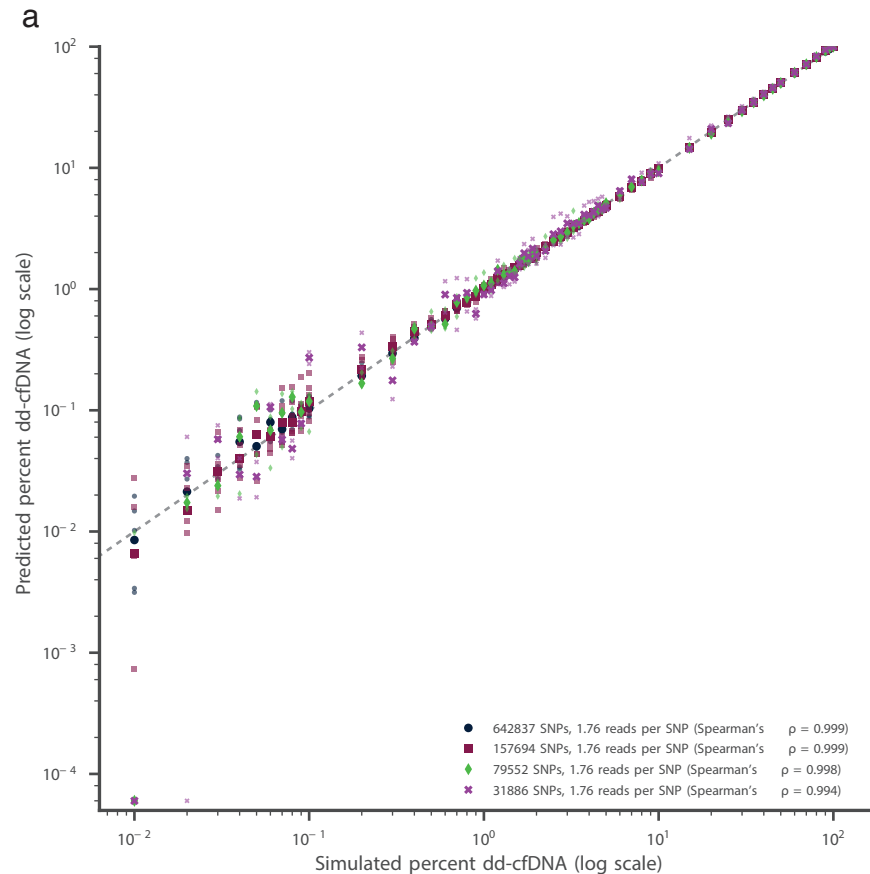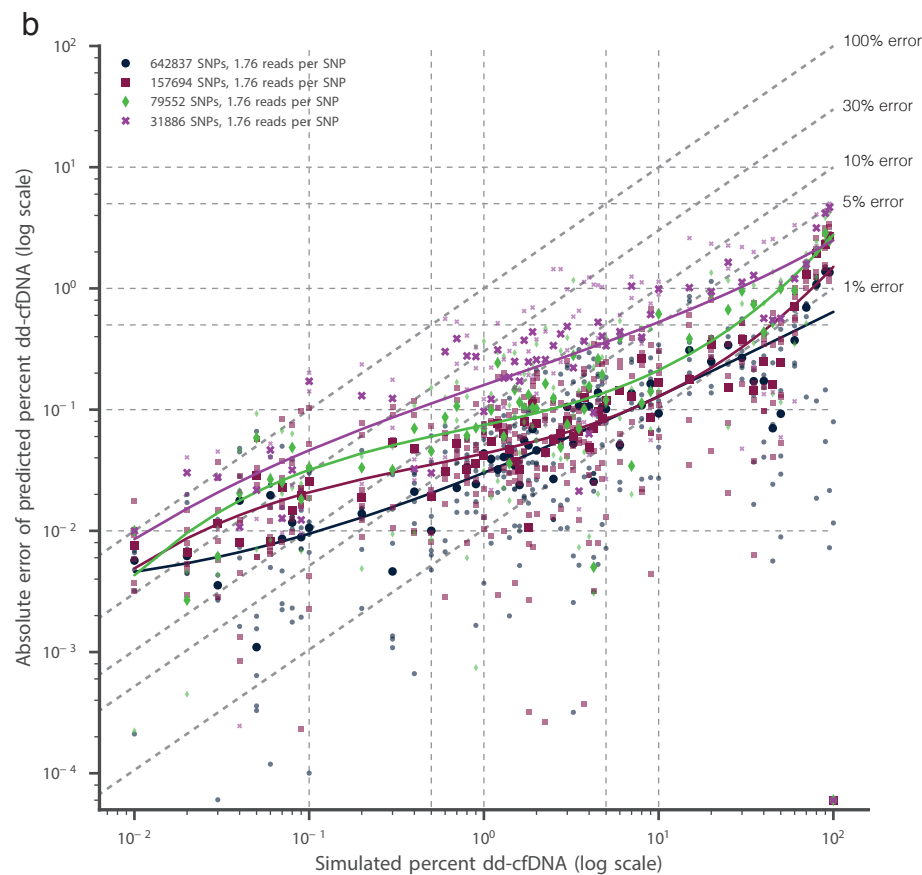

Supplement: S10 Fig — A comparison of the predicted and simulated levels of dd-cfDNA (a) and the corresponding absolute error (b) for different levels of relatedness of the donor and the recipient. dd-cfDNA levels were estimated using 640-700K SNPs and cfDNA sequencing depth of 0.1–1.78x. Larger markers the median result for a simulated dd-cfDNA level, smaller, unconnected markers show independent simulations. Lines show spline interpolation of the median values. (PDF) [file pcbi.1005629.s016.pdf]

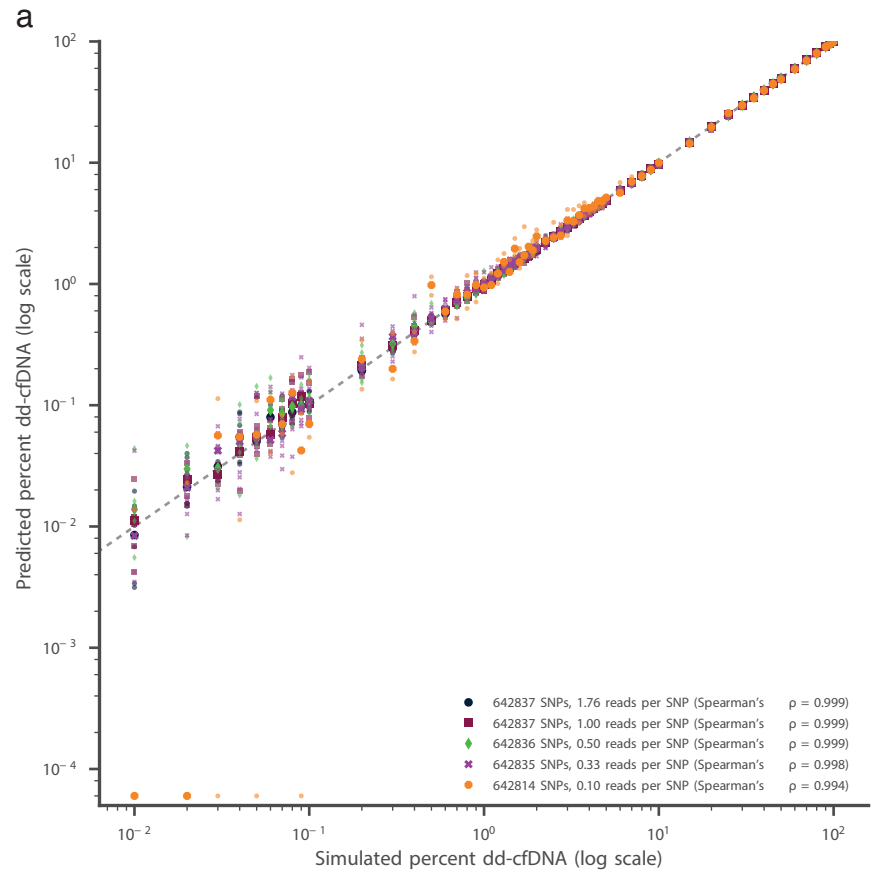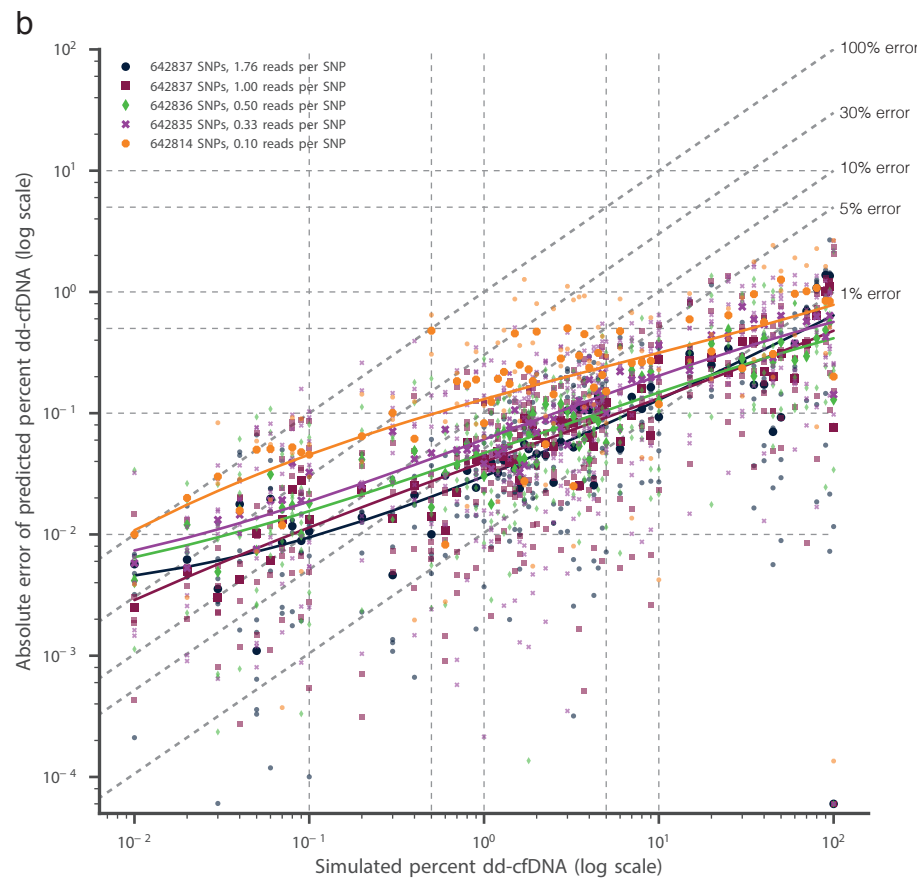

Supplement: S11 Fig — A comparison of the predicted and simulated levels of dd-cfDNA (a) and the corresponding absolute error (b) for different levels of relatedness of the donor and the recipient. dd-cfDNA levels were estimated using 640-700K SNPs and cfDNA sequencing depth of 0.1–1.78x. Larger markers the median result for a simulated dd-cfDNA level, smaller, unconnected marker show independent simulations. Lines show spline interpolation of the median values. (PDF) [file pcbi.1005629.s017.pdf]

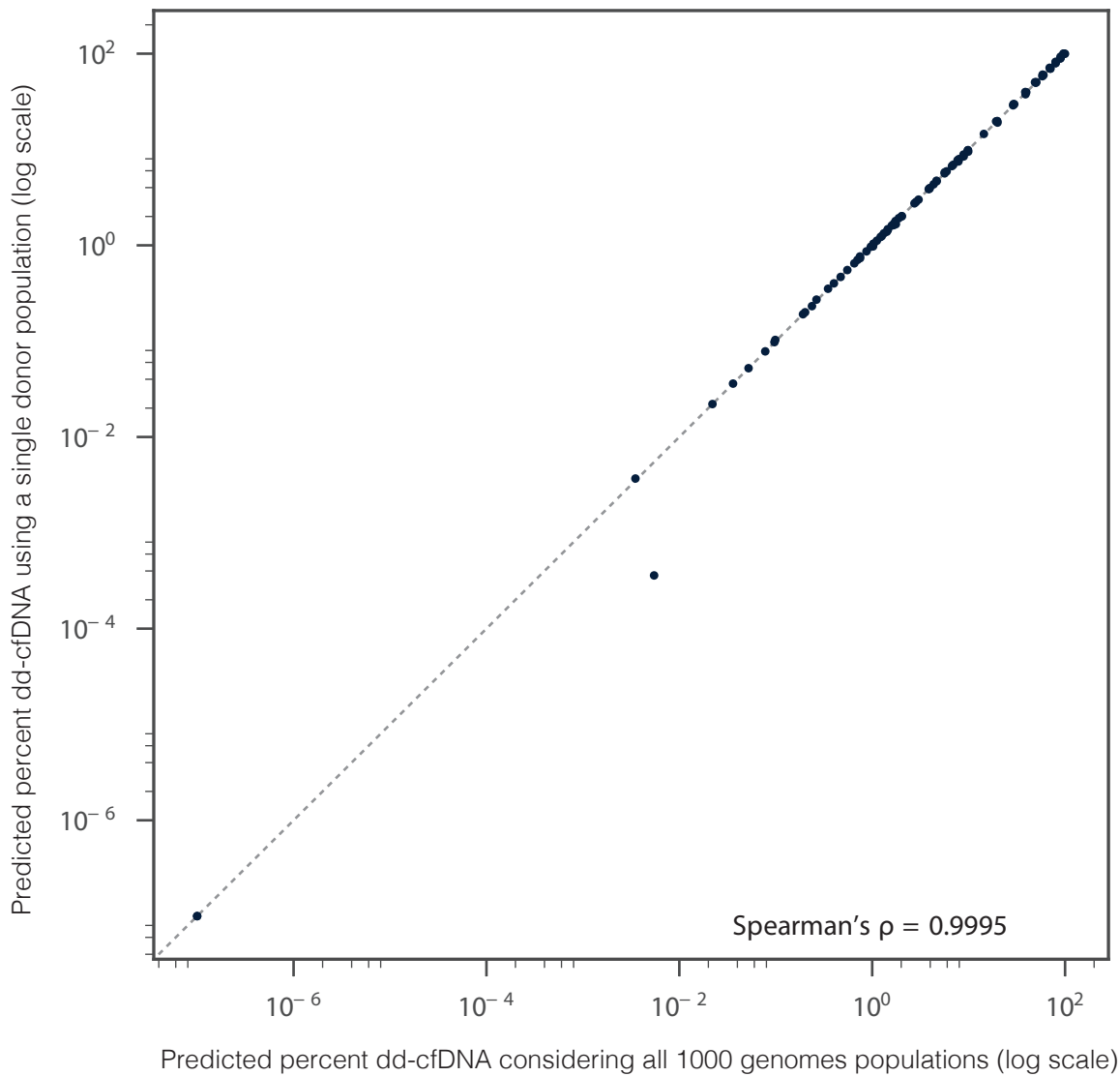

Supplement: S12 Fig — Comparing predicted percent cf-ddDNA using all 1000 genomes project populations (x-axis) to using the single population (y-axis) over 93 simulations (Pearson’s R2 = 0.995). The single population was the population that was inferred for the simulated “donor” sibling in real samples. (PDF) [file pcbi.1005629.s018.pdf]
